# Efficacy and safety of antiviral therapies for the treatment of persistent COVID-19 in immunocompromised patients since the Omicron surge: a systematic review

**DOI:** 10.1093/jac/dkae482

**Published:** 2025-01-13

**Authors:** Caroline Hirsch, Nina Kreuzberger, Nicole Skoetz, Ina Monsef, Stefan Kluge, Christoph D Spinner, Jakob J Malin

**Affiliations:** Institute of Public Health, Faculty of Medicine and University Hospital Cologne, University of Cologne, Kerpener Str. 62, 50939 Cologne, Germany; Institute of Public Health, Faculty of Medicine and University Hospital Cologne, University of Cologne, Kerpener Str. 62, 50939 Cologne, Germany; Institute of Public Health, Faculty of Medicine and University Hospital Cologne, University of Cologne, Kerpener Str. 62, 50939 Cologne, Germany; Institute of Public Health, Faculty of Medicine and University Hospital Cologne, University of Cologne, Kerpener Str. 62, 50939 Cologne, Germany; Department of Intensive Care Medicine, University Medical Center Hamburg-Eppendorf, Martinstr. 52, 20246 Hamburg, Germany; Department of Clinical Medicine, Clinical Department for Internal Medicine II, TUM School of Medicine and Health, University Medical Center, Technical University of Munich, Ismaninger Str. 22, 81675 Munich, Germany; Division of Infectious Diseases, Faculty of Medicine and University Hospital Cologne, Department I of Internal Medicine, University of Cologne, Kerpener Str. 62, 50939 Cologne, Germany

## Abstract

**Background:**

Persistent COVID-19 (pCOVID-19) in immunocompromised patients is characterized by unspecific symptoms and pulmonary infiltrates due to ongoing severe acute respiratory syndrome coronavirus-2 (SARS-CoV-2) replication. Treatment options remain unclear, leading to different approaches, including combination therapy and extended durations. The purpose of this study was to assess the efficacy and safety of antiviral therapies for pCOVID-19 in immunocompromised patients since the Omicron surge.

**Methods:**

We searched MEDLINE and Scopus from 1 January 2022 to 6 August 2024 for cohort studies and case series on nirmatrelvir/ritonavir, remdesivir, ensitrelvir and molnupiravir. Evidence certainty was rated using Grading of Recommendations Assessment, Development, and Evaluation for outcomes including viral clearance, recurrence/relapse, mortality, adverse events (AEs) and symptom resolution.

**Results:**

Thirteen studies involving 127 cases were included. Evidence certainty was very low. In combination therapy with at least two direct antiviral agents, viral clearance was 79%, with a 16% recurrence rate. All-cause mortality was 9%, and mortality was 6% while SARS-CoV-2 positive. In 47 cases, AEs were reported in 11%. Symptom resolution ranged from 3 to 6 days in two studies. In combination therapy with one direct antiviral agent and passive immunization, viral clearance was 89%, with an 11% recurrence rate and no deaths. In four documented cases, no AEs were observed. In monotherapy, viral clearance was 100%, with a 15% recurrence rate. One death, unrelated to SARS-CoV-2, occurred. In 12 documented cases, no AEs were observed.

**Conclusions:**

Based on very low certainty evidence, combining one direct antiviral with passive immunization resulted in high rates of viral clearance and few recurrences. AEs occurred in cases treated with at least two direct antivirals. Controlled studies are needed.

## Introduction

In immunocompromised patients, particularly those with B-cell depletion or residual hypogammaglobulinaemia, prolonged severe acute respiratory syndrome coronavirus-2 (SARS-CoV-2) infections may occur that can persist for several weeks to months. These infections, referred to as persistent COVID-19 (pCOVID-19), are characterized by sustained viral replication in the lower respiratory tract.^1–10^ The clinical presentation of pCOVID-19 significantly differs from acute respiratory infection seen in COVID-19. Affected patients present with general symptoms such as fatigue, weight loss and weakness, subfebrile or febrile temperature or recurrent fever lasting for several weeks to months. Respiratory symptoms such as dry cough and dyspnoea may also be present due to persistent pulmonary ground-glass infiltrates. Since upper respiratory tract symptoms are commonly not prominent in pCOVID-19, the option of a SARS-CoV-2 infection may be neglected in differential diagnostic considerations. Furthermore, PCR tests of the upper respiratory tract are often (intermittently) negative or demonstrate low viral loads, thereby hampering a timely diagnosis of pCOVID-19.^11,12^ Prolonged illness with evidence of ground-glass infiltrates and high SARS-CoV-2 quantitative viral loads in the lower respiratory tract (e.g. detected via bronchoalveolar lavage) are highly suggestive for pCOVID-19. Regarding antiviral therapy, patients with pCOVID-19 require special consideration. First, the general principle that antiviral therapy is only effective in the early phase of infection, before the immune response starts to control viral replication, does not apply. In case of relevant humoral immunodeficiency, endogenous antibody-mediated immune responses may be absent or insufficient to provide a rapid virological control. Therefore, patients with pCOVID-19 may benefit from antiviral treatment irrespective of illness duration. Second, longer treatment durations and/or antiviral combination therapies may be required to achieve control of the viral infection and viral clearance. Some studies show a high rate of persistent or recurrent symptoms and/or ongoing viral shedding indicative of a persistent infection in immunocompromised patients despite completion of standard antiviral therapy courses.^2,3,5,6,13–17^ In addition, immunodeficiency has been identified as potential risk factor for viral rebound after treatment cessation.^18^ Finally, incomplete immunological control of ongoing viral replication potentially occurring under selective pressure from antiviral agents does facilitate the development of resistance-mediating viral mutations. This has been particularly demonstrated for monotherapies with neutralizing monoclonal antibodies.^4,19,20^ While directly acting antivirals are generally considered less prone to resistance development due to their essential intracellular targets, reports on mutation induction are also accumulating for remdesivir^21–24^ and nirmatrelvir^24,25^ in immunocompromised patients treated for prolonged or persistent COVID-19.

In clinical practice, some experts advise to use antiviral combination therapies and/or longer treatment durations compared with those recommended for immunocompetent individuals.^26,27^ However, existing evidence is limited to observational data and current guidelines do not explicitly focus on treatment recommendations for pCOVID-19. This systematic review will review current evidence for efficacy and safety of antiviral treatments in patients with pCOVID-19.

## Methods

### Protocol registration

This systematic review was registered on the International Prospective Register of Systematic Reviews (PROSPERO, identifier: CRD42024556839) and conducted according to the Preferred Reporting Items for Systematic Reviews and Meta-Analysis Statement (PRISMA).^28^ The detailed PRISMA checklist and differences between PROSPERO and the review are given in Appendix Tables S1 and S2 (available as Supplementary data at *JAC* Online).

### Eligibility criteria

#### Types of studies

We included prospective and retrospective cohort studies and case series. We considered studies published as peer-reviewed journal publications. We excluded single case reports and studies published as preprint articles, conference abstracts and results published in study registries.

#### Types of participants

We included immunocompromised adults (≥18 years old) with pCOVID-19 during the Omicron period (starting from1 January 2022), characterized by prolonged viral shedding and persistent or recurring symptomatic SARS-CoV-2 infection, lasting for a minimum of 14 days after disease onset. Individuals were considered immunocompromised if they had one or more of the following immunocompromising conditions previously associated with pCOVID-19: B-cell depleting treatments (previous or active), haematological malignancies (especially chronic lymphocytic leukaemia, multiple myeloma or non-Hodgkin lymphoma), primary or secondary hypogammaglobulinaemia and history of bone marrow, CAR-T cell or solid organ transplantation.^27,29^ Additionally, we included other conditions known to impair humoral immune responses: severe congenital immunodeficiencies, intermittent haemodialysis, solid organ cancers under active chemotherapy, HIV with ≤200 CD4+ cells per millilitre or treatment with highly immunosuppressive medication (e.g. high-dose corticosteroids, methotrexate ≥ 20 mg/week, cyclophosphamide and azathioprine ≥ 3 mg/kg/day).^30^ We limited our search to cases since the beginning of the Omicron period to increase transferability to today’s setting and population in terms of mortality rates and immunity. We included studies irrespective of setting, sex, ethnicity or presence of additional comorbidities.

#### Types of interventions

We included the following direct antiviral agents against SARS-CoV-2 that inhibit viral replication and reduce viral load: nirmatrelvir/ritonavir, remdesivir, ensitrelvir and molnupiravir. These agents were used alone or in combination with another antiviral agent or with a form of passive immunization (SARS-CoV-2-neutralizing monoclonal antibodies or polyclonal immunoglobulins such as intravenous immunoglobulins or convalescent plasma).

All dosing strategies, schedules/frequencies and routes of administration were eligible.

#### Types of outcome measures

We analysed the following outcomes: SARS-CoV-2 viral clearance; COVID-19 recurrence/relapse; all-cause mortality; mortality while SARS-CoV-2 positive; adverse events (AEs); and symptom resolution.

Studies reporting only on risk factors or the frequency of viral shedding were excluded.

### Literature search

We searched MEDLINE and Scopus from 1 January 2022 to 6 August 2024 for nirmatrelvir/ritonavir, remdesivir, ensitrelvir and molnupiravir. The search strategies are provided in Appendix Tables S5–S8.

### Data collection and analysis

#### Selection of studies

The studies were selected using a web-based online platform (Rayyan; https://www.rayyan.ai/).^31^ Two review authors (N.K. and C.H.) independently screened the titles and abstracts resulting from the literature search to identify potentially relevant studies. Subsequently, full-text articles of all potentially relevant records were retrieved and independently assessed for eligibility. Any disagreements between the two authors were resolved through discussion.

#### Data collection

Data extraction was performed by one reviewer (N.K. or C.H.) and verified by another author (N.K. or C.H.) using a standardized data extraction form. We collected data on study characteristics, participant characteristics, interventions, outcome measures, financial support and disclosure of conflicts of interest. In case of missing data, we would have contacted the authors of relevant publications.

#### Risk-of-bias assessment

Two review authors (N.K. and C.H.) independently assessed the risk of bias in the included studies on a per-outcome, per-study basis. Any disagreements between the two authors were resolved through discussion. As we identified only case series, we used an adapted version of the Newcastle–Ottawa scale modified by Cochrane Childhood Cancer.^32^ Refer to Appendix Table S3 for the assessment criteria. For every criterion, we classified the study as having a low, unclear or high risk of bias.

#### Data synthesis

Since we only identified case series, we refrained from conducting meta-analysis, as the value in meta-analysing data that lack a control group may be limited. Instead, we presented the frequencies and percentages of outcome data from each included study in tables and commented on them narratively. As a mix of treatment options was often analysed per cohort, it was not always possible to assign which outcomes occurred under which treatment. Where possible, we have presented the results by treatment approach: monotherapy including a single direct antiviral agent (including those administered sequentially to other antivirals); combination therapy with one direct antiviral agent and passive immunization; and combination therapy with at least two direct antiviral agents (and neutralizing monoclonal antibodies where appropriate).

We performed a post hoc sensitivity analysis comparing combination therapy with polyclonal antibody products with combination therapy with neutralizing monoclonal antibodies. A comparison of different agents was not possible, as many of the included cases received various substances sequentially.

#### Certainty of the evidence

We used the Grading of Recommendations Assessment, Development, and Evaluation (GRADE) approach to assess the certainty in the evidence for all outcomes specified under ‘Types of outcomes measures’, commencing at a low level of evidence for observational studies as per GRADE guidance.^33^ We created separate Summary of Findings tables based on the treatment approaches outlined in the Data synthesis section.

## Results

### Results of the search

**Figure 1.**
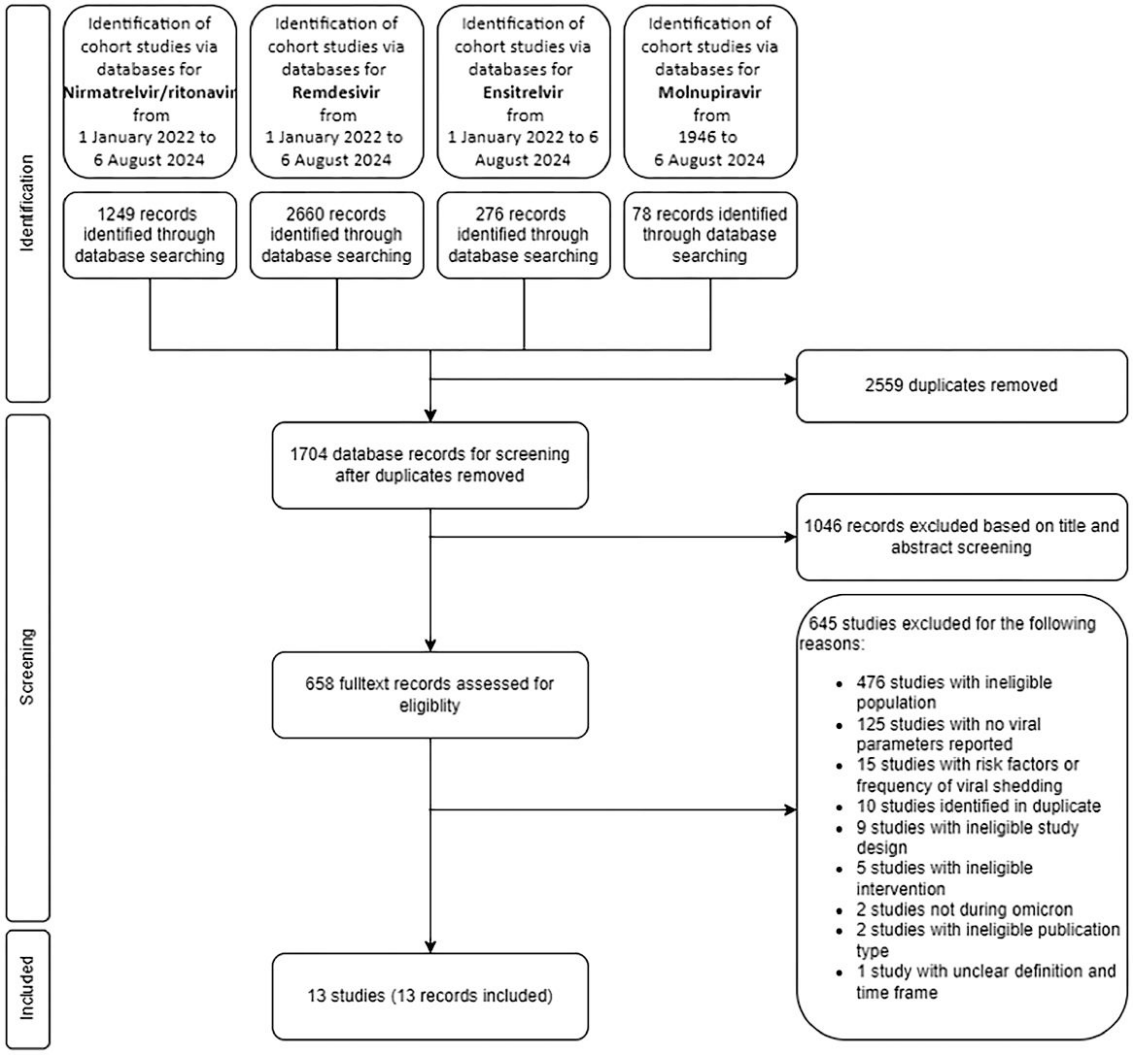
PRISMA flow diagram.

We identified 4263 potentially relevant records. After removing 2559 duplicates, we removed further 1046 records based on their titles and abstracts. Full-text screening of 658 records revealed 645 records that did not meet the inclusion criteria. Although 17 of these studies initially appeared to meet the inclusion criteria, they were excluded because they focused on risk factors or the frequency of viral shedding,^7,9,20,34–46^ or the lack of clarity regarding the definition and timeframe of pCOVID-19.^47^ Finally, we included 13 studies in this review.^2–6,8,11,13–17,48^ The study selection process, along with reasons for exclusion, is illustrated in a PRISMA flow diagram (Figure 1).

### Study characteristics and description of cases

We identified case series or cohort studies that included 127 immunosuppressed cases with pCOVID-19 within their cohort, all lacking a defined control group. These cases received at least one of the following treatments: nirmatrelvir/ritonavir, remdesivir or molnupiravir. No cases treated with ensitrelvir were identified. Detailed information on the characteristics is provided in Table 1 and Appendix Tables S9–S11.

**Table 1.** Characteristics of included studies

Characteristics	Details
Total cases	127 immunosuppressed cases with pCOVID-19
Study type	Case series or cohort studies; all lacking a defined control group
Recruitment period	Most studies during Omicron surge (December 2021–June 2023); one study from May 2020 to December 2023
Funding sources	Six studies funded by academic/government institutions; four without dedicated funding; three undisclosed
Number of cases per study	Ranged from 3 to 15 cases
Geographic distribution of cases	Europe: 82 (64%) Israel: 29 (23%) USA: 15 (12%) China: 6 (5%)
Age range of cases	38–84 years
Proportion of female cases	21%–67%
COVID-19 vaccinated with ≥2 doses (*N* =** **77)	59 (77%)
COVID-19 pre-exposure prophylaxis reported (*N* = 12)	mAbs: 12 (100%), primarily tixagevimab/cilgavimab
Comorbidities reported	Obesity: 5–15% Diabetes: 14–50% Chronic respiratory diseases: 13–21% Hypertension: 57% Chronic kidney disease: 9–29%
Underlying Immunocompromising diseases	Haematological malignancies: 113 (89%) Solid organ transplants: 5 (4%) Autoimmune diseases: 4 (3%) HIV/AIDS: 4 (3%) Other immunodeficiencies: 1 (0.8%)
Concomitant immunosuppressive therapy (*N* =** **94)	B-cell depleting therapies: 68 (72%) Stem cell transplantation/CAR-T cell therapy: 10 (11%) BTK inhibitors: 3 (3%) Others^a^: 6 (6%) None: 7 (7%)
Viral shedding duration	Commonly over 21 days; some cases lasted from 1 to 6 months; maximum duration up to 901 days post-infection onset (not Omicron variant)
Treatment protocol duration for pCOVID-19	Typically lasted 5–10 days
Treatment approaches for pCOVID-19 (*N* =** **127)	At least two DAA: 79: (60%) Two DAA: 36 (59%) Two DAA + mAb: 42 (53%) Three DAA: 1 (1%) DAA + passive immunization: 23 (17%) DAA + intravenous immunoglobulins: 9 (39%) DAA + convalescent plasma: 8 (35%) DAA + mAb: 5 (22%) DAA + convalescent plasma + mAb: 1 (4%) Monotherapy: 23 (18%) Remdesivir: 13 (57%) Nirmatrelvir/ritonavir: 9 (39%) Molnupiravir: 1 (4%) No treatment: 2 (2%)
Previous COVID-19 treatments reported (*N* = 99)	Antiviral monotherapy: 67 (68%) Nirmatrelvir/ritonavir: 32 (48%) Remdesivir: 13 (19%) Molnupiravir: 7 (10%) SARS-CoV-2-neutralizing mAbs: 13 (19%) Convalescent plasma: 2 (3%) One direct antiviral agent + mAbs: 3 (3%) Convalescent plasma + mAbs: 1 (1%) Two direct antiviral agents: 2 (2%) Methylprednisolone: 1 (1%) Not pre-treated: 25 (25%)

BTK, Bruton tyrosine kinase; CAR, chimeric antigen receptor; DAA, direct antiviral agent; mAbs, monoclonal antibodies.

^a^Others include cyclosporine, tacrolimus, everolimus, lenalidomide and mycophenolate mofetil.

### Risk of bias in included studies

The risk of bias was assessed using an adapted version of the Newcastle–Ottawa scale modified by Cochrane Childhood Cancer.^32^ The judgments are presented in Appendix Table S4.

We rated all studies as having a high risk of selection bias due to small sample sizes (3–22 cases) and absence of control groups, which may affect their representativeness. Since all studies were unblinded, we assessed subjective outcomes (e.g. COVID-19 recurrence/relapse, AEs and symptom resolution) with a high risk of detection bias. We judged attrition bias to be low as outcome data were complete for individual cases.

We assessed reporting bias based on well-defined study groups and interventions, and outcomes. Three studies^4,15,17^ did not report the characteristics of cases with pCOVID-19, leading to an unclear risk of bias. The other studies were judged to be of low risk of reporting bias. In terms of outcome definitions, all studies provided clear definitions, resulting in a low risk of bias. Furthermore, we were unable to assess the risk of confounding, as no comparison and effect estimators were available.

### Efficacy and safety of antiviral therapies

**Figure 2.**
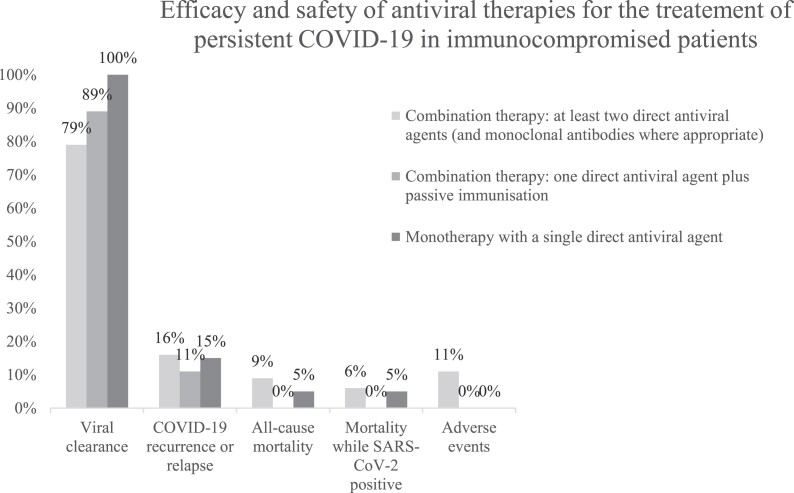
Bar chart of outcome data.

The findings from the analysis of treatment outcomes for patients with pCOVID-19 are summarized in Tables 2–4, Figure 2 and Appendix Tables S12–S14. These tables present data categorized into three different treatment approaches in the order of the most evidence available: combination therapy with at least two direct antiviral agents and neutralizing monoclonal antibodies where appropriate, combination therapy with one direct antiviral agent and any passive immunization and monotherapy involving a single direct antiviral agent (including those administered sequentially to other antivirals). The overall certainty in the evidence is rated as very low across all outcomes due to significant limitations in study design and imprecision due to small sample sizes.

**Table 2.** Summary of findings: combination therapy with at least two direct antiviral agents (and neutralizing monoclonal antibodies where appropriate)

Outcome	Certainty assessment						Summary of findings		
	Study design	Risk of bias	Inconsistency	Indirectness	Imprecision	Publication bias	No. of cases (studies)	Narrative outcome	Certainty
Viral clearance	Case series^a^	Very serious^a^	No serious	No serious	Serious^b^	No serious	67 (7 studies^2,3,5,11,14,15,48^)	Viral clearance was observed in 53 out of 67 cases (79%).	⊕⊖⊖⊖ Very low
COVID-19 recurrence/relapse	Case series^a^	Very serious^a^	No serious	No serious	Serious^b^	No serious	67 (7 studies^2,3,5,11,14,15,48^)	COVID-19 recurrence/relapse was observed in 11 out of 67 cases (16%).	⊕⊖⊖⊖ Very low
All-cause mortality	Case series^a^	Very serious^a^	No serious	No serious	Serious^b^	No serious	67 (7 studies^2,3,5,11,14,15,48^)	All-cause mortality was observed in six out of 67 cases (9%).	⊕⊖⊖⊖ Very low
Mortality while SARS-CoV-2 positive	Case series^a^	Very serious^a^	No serious	No serious	Serious^b^	No serious	67 (7 studies^2,3,5,11,14,15,48^)	Mortality while SARS-CoV-2 positive was observed in four out of 67 cases (6%).	⊕⊖⊖⊖ Very low
AEs	Case series^a^	Very serious^a^	No serious	No serious	Serious^b^	No serious	47 (4 studies^2,3,5,48^)	AEs were observed in five out of 47 cases (11%).	⊕⊖⊖⊖ Very low
Symptom resolution	Case series^a^	Very serious^a^	No serious	No serious	Serious^b^	No serious	19 (2 studies^2,14^)	One study reported a median time to symptom resolution of 3 (IQR 1–3) days for 15 cases in one study, while the other study reported a median time of 6 days (IQR 4.2–10.7) for 14 cases.	⊕⊖⊖⊖ Very low

Certainty of the evidence: ⊕⊕⊕⊕ high; ⊕⊕⊕⊖ moderate; ⊕⊕⊖⊖ low; ⊕⊖⊖⊖ very low.

^a^Started with low level of evidence due to very serious limitations in study design according to GRADE guidance.^33^

^b^Downgraded one level due to serious imprecision caused by extremely small sample sizes and the use of different treatment options sequentially, which made it impossible to pool results and compare between different treatment options.

**Table 3.** Summary of findings: combination therapy with one direct antiviral agent and passive immunization

Outcome	Certainty assessment						Summary of findings		
	Study design	Risk of bias	Inconsistency	Indirectness	Imprecision	Publication bias	No. of cases (studies)	Narrative outcome	Certainty
Viral clearance	Case series^a^	Very serious^a^	No serious	No serious	Serious^b^	No serious	19 (4 studies^5,6,13,17^)	Viral clearance was observed in 17 out of 19 cases (89%).	⊕⊖⊖⊖ Very low
COVID-19 recurrence/relapse	Case series^a^	Very serious^a^	No serious	No serious	Serious^b^	No serious	19 (4 studies^5,6,13,17^)	COVID-19 recurrence/relapse was observed in two out of 19 (11%).	⊕⊖⊖⊖ Very low
All-cause mortality	Case series^a^	Very serious^a^	No serious	No serious	Serious^b^	No serious	19 (4 studies^5,6,13,17^)	All-cause mortality was observed in none of the 19 cases.	⊕⊖⊖⊖ Very low
Mortality while SARS-CoV-2 positive	Case series^a^	Very serious^a^	No serious	No serious	Serious^b^	No serious	19 (4 studies^5,6,13,17^)	Mortality while SARS-CoV-2 positive was observed in none of the 19 cases.	⊕⊖⊖⊖ Very low
AEs	Case series^a^	Very serious^a^	No serious	No serious	Serious^b^	No serious	4 (2 studies^5,17^)	AEs were observed in none of the four cases.	⊕⊖⊖⊖ Very low
Symptom resolution	—	—	—	—	—	—	—	We did not identify any study reporting symptom resolution for cases with pCOVID-19 treated with monotherapy.	—

Certainty of the evidence: ⊕⊕⊕⊕ high; ⊕⊕⊕⊖ moderate; ⊕⊕⊖⊖ low; ⊕⊖⊖⊖ very low.

^a^Started with low level of evidence due to very serious limitations in study design according to GRADE guidance.^33^

^b^Downgraded one level due to serious imprecision caused by extremely small sample sizes and the use of different treatment options sequentially, which made it impossible to pool results and compare between different treatment options.

**Table 4.** Summary of findings: Monotherapy including a single direct antiviral agent

Outcome	Certainty assessment						Summary of findings		
	Study design	Risk of bias	Inconsistency	Indirectness	Imprecision	Publication bias	No. of cases (studies)	Narrative outcome	Certainty
Viral clearance	Case series^a^	Very serious^a^	No serious	No serious	Serious^b^	No serious	20 (3 studies^5,11,16^)	Viral clearance was observed in 20 out of 20 cases (100%)	⊕⊖⊖⊖ Very low
COVID-19 recurrence/relapse	Case series^a^	Very serious^a^	No serious	No serious	Serious^b^	No serious	20 (3 studies^5,11,16^)	COVID-19 recurrence/relapse was observed in three out of 20 cases (15%)	⊕⊖⊖⊖ Very low
All-cause mortality	Case series^a^	Very serious^a^	No serious	No serious	Serious^b^	No serious	20 (3 studies^5,11,16^)	All-cause mortality was observed in one out of 20 cases (5%) with pCOVID-19 treated with monotherapy	⊕⊖⊖⊖ Very low
Mortality while SARS-CoV-2 positive	Case series^a^	Very serious^a^	No serious	No serious	Serious^b^	No serious	20 (3 studies^5,11,16^)	Mortality while SARS-CoV-2 positive was observed in one out of 20 cases (5%)	⊕⊖⊖⊖ Very low
AEs	Case series^a^	Very serious^a^	No serious	No serious	Serious^b^	No serious	12 (1 study^5^)	No AEs were documented in any of the 12 cases	⊕⊖⊖⊖ Very low
Symptom resolution	—	—	—	—	—	—	—	We did not identify any study reporting symptom resolution for cases with pCOVID-19 treated with monotherapy	—

Certainty of the evidence: ⊕⊕⊕⊕ high; ⊕⊕⊕⊖ moderate; ⊕⊕⊖⊖ low; ⊕⊖⊖⊖ very low.

^a^Started with low level of evidence due to very serious limitations in study design according to GRADE guidance.^33^

^b^Downgraded one level due to serious imprecision caused by extremely small sample sizes and the use of different treatment options sequentially, which made it impossible to pool results and compare between different treatment options.

#### Combination therapy with at least two direct antiviral agents (and neutralizing monoclonal antibodies where appropriate)

Among 67 cases reported across seven studies, viral clearance was achieved in 53 cases (79%). However, COVID-19 recurrence or relapse was observed in 11 out of 67 cases (16%) and required one or more additional treatment courses to eliminate the virus. All-cause mortality was observed in six out of 67 cases (9%), with four deaths occurring while the cases were still positive for SARS-CoV-2 or died due to COVID-19. AEs were reported in five out of 47 cases (11%). These events included nausea and/or dysgeusia in two cases (4%) receiving a regimen containing nirmatrelvir/ritonavir,^48^ myocardial infarction in one case (2%) and asymptomatic sinus bradycardia in another case (2%) after initiation of a combination therapy including two direct antiviral agents.^3^ One case (2%) treated with remdesivir and nirmatrelvir/ritonavir experienced severe AEs due to suspected hepatotoxicity.^5^ Two studies reported on symptom resolution; one study indicated a median time to symptom resolution of 3 days (IQR 1–3) for 15 cases,^14^ while another study reported a median time of 6 days (IQR 4.2–10.7) for 14 cases.^15^

Out of the 67 cases, 27 (40%) received a combination of two direct antivirals along with neutralizing monoclonal antibodies, specifically 78% tixagevimab/cilgavimab and 22% sotrovimab.^3,14,48^ The remaining 40 cases (60%) received a combination of two direct antivirals.^2,3,5,11,14,15,48^

#### Combination therapy with one direct antiviral agent and passive immunization

Among 19 cases reported across four studies, viral clearance was achieved in 17 cases (89%). Two out of 19 cases (11%) experienced COVID-19 recurrence or relapse. None of the 19 cases died. Among four cases reported across two studies, no AEs were documented in any of the cases. No studies were identified that reported on symptom resolution.

Out of the 19 cases, 17 (90%) received a combination of a direct antiviral agent and a polyclonal antibody product [intravenous immunoglobulins (47%), convalescent plasma (32%) and hyperimmune plasma (11%)].^6,13,17^ In 10% of the cases, the direct antiviral agents were combined with neutralizing monoclonal antibodies, either alone (5%) or in combination with a polyclonal antibody product (5%).^5,17^ A sensitivity analysis comparing combination therapy with polyclonal antibody products to combination therapy with neutralizing monoclonal antibodies (see Appendix Table S15) showed that the virus was successfully cleared in all cases receiving monoclonal antibodies (2/2), with no recurrences or relapses (0/2). In cases receiving combination therapy with polyclonal antibody products, viral clearance occurred in 88% (15/17) of cases, with a recurrence rate of 12% (2/17). There were no deaths in either group.

#### Monotherapy including a single direct antiviral agent

Among 20 cases across three studies, viral clearance was achieved in all 20 cases (100%). Three out of the 20 cases (15%) experienced recurrence or relapse after treatment and required one or more additional treatment courses to eliminate the virus. One case died while still SARS-CoV-2 positive due to causes unrelated to SARS-CoV-2. Among 12 cases reported across one study, no AEs were documented in any of the cases. No studies were identified that reported on symptom resolution. All cases were treated with a single direct antiviral agent, which in some cases was administered sequentially with other antivirals^5,11,16^

## Discussion

### Summary of the main results

This systematic review identified 13 case series or cohort studies involving a total of 127 immunosuppressed cases with pCOVID-19 during the Omicron surge, all without controls, and receiving treatment with at least one of the following drugs: nirmatrelvir/ritonavir, remdesivir or molnupiravir. Various underlying conditions were present among the cases, including B-cell-depleting therapies, haematological malignancies, hypogammaglobulinaemia, solid organ transplantation and other relevant immunodeficiencies. Most cases were treated with a combination of at least two direct antiviral agents. In these cases, viral clearance was achieved in 79% of cases, with a 16% recurrence rate and a 9% all-cause mortality rate, alongside reported AEs in 11% of cases, mainly associated with nirmatrelvir/ritonavir. While hepatotoxicity is a potential side effect associated with nirmatrelvir/ritonavir, our findings indicate that it is relatively uncommon, with only one out of 47 cases reporting related AEs. Regular monitoring of liver function during treatment may be advisable to ensure patient safety and address any emerging concerns. Combination therapies with one direct antiviral agent and passive immunization resulted in an 89% viral clearance rate, a recurrence rate of 11% and no reported deaths among the cases analysed. Monotherapy including a single direct antiviral agent achieved viral clearance in all cases, but had a 15% recurrence rate and one death unrelated to SARS-CoV-2 among the cases treated.

Overall, the evidence regarding the effectiveness and safety of antiviral therapies for pCOVID-19 in immunocompromised patients remains uncertain across all three treatment approaches due to very low certainty evidence from the included studies. Although the findings are based on case series only, treatment approaches with combination therapy may offer potential benefits in achieving SARS-CoV-2 clearance and reducing the risk of recurrence in immunocompromised patients with persistent SARS-CoV-2 infection. While monotherapy with antiviral agents demonstrated a complete clearance rate, it was also associated with a slightly higher recurrence rate. Of note, a substantial proportion of identified cases with pCOVID-19 (68%, 67/99) were pre-treated with antiviral monotherapy supporting a relatively high rate of treatment failure in the immunocompromised patient population described. Further research, particularly prospective controlled and comparative studies, is needed to establish more definitive conclusions on the effectiveness and safety of different antiviral treatment approaches in this specific patient population. The findings of the present review were used to inform a recommendation for the German living evidence-based guideline for the treatment of patients with COVID-19.^49^

### Agreements and disagreements with other studies or reviews

In a consensus statement, the Israeli Society of Infectious Diseases recommends a combination therapy consisting of antibody-based therapy and two antiviral drugs for 5–10 days for the treatment of pCOVID-19.^27^ The statement was also based on very low certainty evidence from case reports and case series. Unlike in the present review, cases were not limited to the Omicron period and single case reports were included.

### Strengths and limitations

This systematic review was registered with PROSPERO and adhered to the PRISMA guidance.^28^ The search strategies were developed by an experienced information specialist, who conducted a sensitive search across relevant databases to ensure comprehensive results. Search results were screened by two reviewers independently. Data extraction and risk-of-bias assessments were double-checked by a second reviewer.

The evidence has limitations, including a high risk of bias in the included studies, which were case series with very small sample sizes and non-random sampling. Additionally, we were not able to compare more detailed differences in treatment approaches such as different sequential strategies, timings and treatment durations due to small and heterogenic groups reported retrospectively from clinical observations. The lack of data from randomized trials including a control group hinders drawing meaningful conclusions from the results.

In terms of the review process, due to the rarity of persistent shedding, we did not expect randomized controlled trials and limited the search to cohort studies and case series. Furthermore, we did not define specific time frames for the outcomes analysed and instead, used the time frames reported in the studies. The studies reported outcomes at heterogeneous time points; for instance, one study reported mortality at 90 days,^5^ while another study reported it at 60 days.^3^ As a result, attributing these outcomes directly to COVID-19 may be challenging.

### Conclusions

pCOVID-19 is a distinct condition most commonly affecting immunocompromised patients, particularly with B-cell depletion. Most reported cases experienced pCOVID-19 despite one or multiple treatment attempts with antiviral monotherapy, indicating that intensified treatment approaches may be warranted. Our findings suggest that antiviral combination therapies may provide potential benefits in managing pCOVID-19 although evidence is limited to observational data from case series and comparative data are lacking. Based on very low certainty evidence, combination therapy with one direct acting antiviral and passive immunization resulted in relatively few recurrences compared with monotherapy or combination of at least two direct acting antivirals. Overall, most reported AEs occurred in patients with pCOVID-19 treated with at least two different direct acting antivirals and included one severe event of hepatotoxicity. Further research, particularly controlled studies, is needed to establish more definitive conclusions on the efficacy and safety of these treatments.

## Supplementary Material

dkae482_Supplementary_Data

## Transparency declarations

S.K. received research support from Cytosorbents and Daiichi Sankyo. He also received lecture fees from ADVITOS, Biotest, Daiichi Sankyo, Fresenius Medical Care, Gilead, Mitsubishi Tanabe Pharma, MSD, Pfizer, Shionogi and Zoll. He received consultant fees from ADVITOS, Fresenius, Gilead, MSD and Pfizer. C.D.S. reports grants, personal fees from AstraZeneca, personal fees and non-financial support from BBraun Melsungen, personal fees from BioNtech, grants, personal fees and non-financial support from Bavarian Nordic, personal fees and non-financial support from Gilead Sciences, grants and personal fees from Janssen-Cilag, personal fees from Eli Lilly, personal fees from Formycon, personal fees from Pfizer, personal fees from Roche, other from Apeiron, grants and personal fees from MSD, grants from Cepheid, personal fees from GSK, personal fees from Molecular partners, personal fees from Pfizer, other from Eli Lilly, personal fees from SOBI during the conduct of the study; personal fees from AbbVie, personal fees from MSD, personal fees from Synairgen, personal fees from Shionogi and grants and personal fees from ViiV Healthcare, outside the submitted work. J.J.M. received consulting honoraria and/or speaker’s fees from Astra Zeneca, Gilead Sciences, Merck Sharp & Dohme and Janssen Cilag. All other authors: none to declare.

## Supplementary data

Tables S1–S15 are available as Supplementary data at *JAC* Online.
