# Supplementary material for: Efficacy and safety of antiviral therapies for the treatment of persistent COVID-19 in immunocompromised patients since the Omicron surge: a systematic review
Source: J Antimicrob Chemother. 2025 Jan 13;80(3):633–44. doi: 10.1093/jac/dkae482 (PMC11879234; doi:10.1093/jac/dkae482)
Supplement: dkae482_Supplementary_Data [file dkae482_supplementary_data.docx]

**Appendix**

**Efficacy and safety of antiviral therapies for the treatment of persistent COVID-19 in immunocompromised patients since the Omicron surge: a systematic review**

Caroline HIRSCH^1*^, Nina KREUZBERGER^1^, Nicole SKOETZ^1^, Ina MONSEF^1^, Stefan KLUGE^2^, Christoph D. SPINNER^3^, and Jakob J. MALIN^4^

^1^Institute of Public Health, Faculty of Medicine and University Hospital Cologne, University of Cologne, Cologne, Germany

^2^Department of Intensive Care Medicine, University Medical Center Hamburg-Eppendorf, Hamburg, Germany

^3^TUM School of Medicine and Health, Department of Clinical Medicine – Clinical Department for Internal Medicine II, University Medical Center, Technical University of Munich, Munich, Germany

^4^Department I of Internal Medicine, Division of Infectious Diseases, Faculty of Medicine and University Hospital Cologne, University of Cologne, Cologne, Germany

**Corresponding author**

Name: Caroline Hirsch

Address: Kerpener Str. 62, 50939 Cologne, Germany

E-Mail: [caroline.hirsch@uk-koeln.de](mailto:caroline.hirsch@uk-koeln.de)

Phone: +49 221 478 62032

**Contents**

Table S1: PRISMA 2020 checklist

Table S2: Differences between protocol (PROSPERO) and review

Table S3: Risk of bias assessment criteria for observational studies

Table S4: Risk of bias assessments

Table S5: Search strategies nirmatrelvir/ritonavir

Table S6: Search strategies remdesivir

Table S7: Search strategies ensitrelvir

Table S8: Search strategies molnupiravir

Table S9: Study characteristics

Table S10: Participant characteristics

Table S11: Characteristics of viral persistence

Table S12: Outcomes - combination therapy with at least two direct antiviral agents

Table S13: Outcomes - combination therapy with one direct antiviral agent and passive immunisation

Table S14: Outcomes - monotherapy including a single direct antiviral agent

Table S15: Sensitivity analysis: combination therapy with one direct antiviral agent and polyclonal antibodies versus combination therapy with one direct antiviral agent and monoclonal antibodies

**Table S1: PRISMA checklist**

| **Section and Topic** | **Item #** | **Checklist item** | **Location where item is reported** |
| --- | --- | --- | --- |
| **TITLE** | | |  |
| Title | 1 | Identify the report as a systematic review. | Title |
| **ABSTRACT** | | |  |
| Abstract | 2 | See the PRISMA 2020 for Abstracts checklist. | Abstract |
| **INTRODUCTION** | | |  |
| Rationale | 3 | Describe the rationale for the review in the context of existing knowledge. | Introduction |
| Objectives | 4 | Provide an explicit statement of the objective(s) or question(s) the review addresses. | Introduction |
| **METHODS** | | |  |
| Eligibility criteria | 5 | Specify the inclusion and exclusion criteria for the review and how studies were grouped for the syntheses. | Methods – Eligibility criteria |
| Information sources | 6 | Specify all databases, registers, websites, organisations, reference lists and other sources searched or consulted to identify studies. Specify the date when each source was last searched or consulted. | Methods – Literature search |
| Search strategy | 7 | Present the full search strategies for all databases, registers and websites, including any filters and limits used. | Tables S5-S8, Appendix 1 |
| Selection process | 8 | Specify the methods used to decide whether a study met the inclusion criteria of the review, including how many reviewers screened each record and each report retrieved, whether they worked independently, and if applicable, details of automation tools used in the process. | Methods – Selection of studies |
| Data collection process | 9 | Specify the methods used to collect data from reports, including how many reviewers collected data from each report, whether they worked independently, any processes for obtaining or confirming data from study investigators, and if applicable, details of automation tools used in the process. | Methods – Data collection |
| Data items | 10a | List and define all outcomes for which data were sought. Specify whether all results that were compatible with each outcome domain in each study were sought (e.g. for all measures, time points, analyses), and if not, the methods used to decide which results to collect. | Methods – Types of outcomes measures |
|  | 10b | List and define all other variables for which data were sought (e.g. participant and intervention characteristics, funding sources). Describe any assumptions made about any missing or unclear information. | Methods – Data collection |
| Study risk of bias assessment | 11 | Specify the methods used to assess risk of bias in the included studies, including details of the tool(s) used, how many reviewers assessed each study and whether they worked independently, and if applicable, details of automation tools used in the process. | Methods – Risk of bias assessment |
| Effect measures | 12 | Specify for each outcome the effect measure(s) (e.g. risk ratio, mean difference) used in the synthesis or presentation of results. | Methods – Data synthesis |
| Synthesis methods | 13a | Describe the processes used to decide which studies were eligible for each synthesis (e.g. tabulating the study intervention characteristics and comparing against the planned groups for each synthesis (item #5)). | Methods – Data synthesis |
|  | 13b | Describe any methods required to prepare the data for presentation or synthesis, such as handling of missing summary statistics, or data conversions. | NA |
|  | 13c | Describe any methods used to tabulate or visually display results of individual studies and syntheses. | Methods – Certainty of the evidence |
|  | 13d | Describe any methods used to synthesize results and provide a rationale for the choice(s). If meta-analysis was performed, describe the model(s), method(s) to identify the presence and extent of statistical heterogeneity, and software package(s) used. | NA |
|  | 13e | Describe any methods used to explore possible causes of heterogeneity among study results (e.g. subgroup analysis, meta-regression). | NA |
|  | 13f | Describe any sensitivity analyses conducted to assess robustness of the synthesized results. | NA |
| Reporting bias assessment | 14 | Describe any methods used to assess risk of bias due to missing results in a synthesis (arising from reporting biases). | NA |
| Certainty assessment | 15 | Describe any methods used to assess certainty (or confidence) in the body of evidence for an outcome. | Methods – Certainty of the evidence |
| **RESULTS** | | |  |
| Study selection | 16a | Describe the results of the search and selection process, from the number of records identified in the search to the number of studies included in the review, ideally using a flow diagram. | Results of the search, Figure 1 |
|  | 16b | Cite studies that might appear to meet the inclusion criteria, but which were excluded, and explain why they were excluded. | Results of the search |
| Study characteristics | 17 | Cite each included study and present its characteristics. | Table 1, Tables S9-S11 |
| Risk of bias in studies | 18 | Present assessments of risk of bias for each included study. | Results – Risk of bias in included studies, Table S4 |
| Results of individual studies | 19 | For all outcomes, present, for each study: (a) summary statistics for each group (where appropriate) and (b) an effect estimate and its precision (e.g. confidence/credible interval), ideally using structured tables or plots. | Results – Tables 2-4 |
| Results of syntheses | 20a | For each synthesis, briefly summarise the characteristics and risk of bias among contributing studies. | Results – Tables 2-4 |
|  | 20b | Present results of all statistical syntheses conducted. If meta-analysis was done, present for each the summary estimate and its precision (e.g. confidence/credible interval) and measures of statistical heterogeneity. If comparing groups, describe the direction of the effect. | NA |
|  | 20c | Present results of all investigations of possible causes of heterogeneity among study results. | NA |
|  | 20d | Present results of all sensitivity analyses conducted to assess the robustness of the synthesized results. | NA |
| Reporting biases | 21 | Present assessments of risk of bias due to missing results (arising from reporting biases) for each synthesis assessed. | NA |
| Certainty of evidence | 22 | Present assessments of certainty (or confidence) in the body of evidence for each outcome assessed. | Results – Tables 2-4 |
| **DISCUSSION** | | |  |
| Discussion | 23a | Provide a general interpretation of the results in the context of other evidence. | Discussion- Summary of main results |
|  | 23b | Discuss any limitations of the evidence included in the review. | Discussion – Strengths and limitations |
|  | 23c | Discuss any limitations of the review processes used. | Discussion – Strengths and limitations |
|  | 23d | Discuss implications of the results for practice, policy, and future research. | Conclusion |
| **OTHER INFORMATION** | | |  |
| Registration and protocol | 24a | Provide registration information for the review, including register name and registration number, or state that the review was not registered. | Methods – Protocol registration |
|  | 24b | Indicate where the review protocol can be accessed, or state that a protocol was not prepared. | Methods – Protocol registration |
|  | 24c | Describe and explain any amendments to information provided at registration or in the protocol. | Methods – Protocol registration |
| Support | 25 | Describe sources of financial or non-financial support for the review, and the role of the funders or sponsors in the review. | Funding |
| Competing interests | 26 | Declare any competing interests of review authors. | Transparency declarations |
| Availability of data, code and other materials | 27 | Report which of the following are publicly available and where they can be found: template data collection forms; data extracted from included studies; data used for all analyses; analytic code; any other materials used in the review. | Data extracted from included studies in Table 1 and Tables S12-S14 |

**Table S2: Differences between protocol (PROSPERO) and review**

|  | Protocol stage | Full review | Reasons |
| --- | --- | --- | --- |
| Review question | To assess the efficacy and safety of antiviral combination therapies for the treatment of persistent COVID-19 in immunocompromised patients during the Omicron period. | To assess the efficacy and safety of antiviral therapies for the treatment of persistent COVID-19 in immunocompromised patients during the Omicron period. | For clarification, combination therapies were removed. We included cases that received minimum treatment with nirmatrelvir/ritonavir or remdesivir or ensitrelvir or molnupiravir. We also included combination therapies but did not explicitly search for them. |
| Participants/population | Inclusion criteria: studies on immunocompromised adults (≥ 18 years old), with persistent COVID-19 during the Omicron period (starting from 01.01.2022), characterized by prolonged viral shedding and persistent or recurring symptomatic SARS-CoV-2 infection, for example lasting for a minimum of 14 days after disease onset. | We included immunocompromised adults (≥ 18 years old) with pCOVID-19 during the Omicron period (starting from 01.01.2022), characterised by prolonged viral shedding and persistent or recurring symptomatic SARS-CoV-2 infection, lasting for a minimum of 14 days after disease onset. | To enhance clarity and ensure that our analysis specifically targets pCOVID rather than just immunocompromised patients with acute COVID-19, we explicitly defined pCOVID as requiring a minimum duration of 14 days of viral shedding or persistent symptoms after disease onset. |
| Intervention | We included antiviral COVID-19 drugs that inhibit viral replication and were authorised for use in the European Union by the European Medicines Agency (EMA). As of 1 June 2024, those were:   - Nirmatrelvir/ritonavir alone or in combination with any antiviral/ SARS-CoV-2-neutralising monoclonal antibodies (mAbs) or polyclonal immunoglobulins (intravenous immunoglobulins or convalescent plasma) - Remdesivir alone or in combination with any antiviral/ mAbs or polyclonal immunoglobulins (intravenous immunoglobulins (IVIG) or convalescent plasma). | We included the following direct antiviral agents against SARS-CoV-2 that inhibit viral replication and reduce viral load: nirmatrelvir/ritonavir, remdesivir, ensitrelvir molnupiravir. These agents were used alone or in combination with another antiviral agent or with a form of passive immunisation (SARS-CoV-2-neutralising monoclonal antibodies, or polyclonal immunoglobulins such as intravenous immunoglobulins or convalescent plasma). | This systematic review was conducted to inform the German evidence-based guideline for treatment of patients with COVID-19. Ensitrelvir and molnupiravir were not relevant for this guideline. However, ensitrelvir and molnupiravir are of international importance internationally and were therefore subsequently included. |
| Publication format | We will consider:  - Peer-reviewed journal publications  - Preprint articles  - Results published in trial registries  - Conference abstracts | We excluded preprint articles, conference abstracts and results published in study registries. | So that the findings of the review can be used to inform a recommendation in a guideline. |
| Data synthesis | No sensitivity analysis planned | We performed a post-hoc sensitivity analysis comparing combination therapy with polyclonal antibody products to combination therapy with neutralising monoclonal antibodies | Given the potential clinical significance of understanding the differences between monoclonal antibodies and polyclonal antibodies derived from plasma preparations, we believe that conducting separate assessments of these therapies could yield valuable insights for clinicians. Therefore, we decided to perform a post-hoc sensitivity analysis to explore how combination therapies involving these different types of antibody treatments may impact patient outcomes. |

**Table S2: Risk of bias assessment criteria for observational studies**

|  | Internal validity | External validity |
| --- | --- | --- |
| Study group | Selection bias (representative: yes/no)   - if the described study group consisted of > 80% of immunocompromised individuals with persistent COVID-19 treated with antiviral combination therapy in the original cohort - ***or***if it was a random sample concerning the treatment and important prognostic factors | Reporting bias (well defined: yes/no)   - if the study population is well described (e.g. age, comorbidities, co-interventions) - ***and***if the intervention is well described (e.g. antiviral regimen, duration of treatment |
| Follow-up | Attrition bias (adequate: yes/no)   - if the outcome was assessed for > 90% of the study group of interest (++) - ***or***if the outcome was assessed for 60% to 90% of the study group of interest (+) | Reporting bias (well defined: yes/no)   - if the length of follow‐up was mentioned |
| Outcomes | Detection bias (blind: yes/no)   - if the outcome assessors were blinded to the investigated determinant | Reporting bias (well‐defined: yes/no)   - if the outcome definition was objective and precise |
| Risk estimation | Confounding (adjustment for other factors: yes/no)   - if important prognostic factors (i.e. age, gender, co‐treatment) or follow‐up were taken adequately into account | Analyses (well‐defined: yes/no)   - if a relative risk, odds ratio, attributable risk, linear or logistic regression model, mean difference or Chi^2^ was calculated |

**Table S3: Risk of bias assessments**

|  | Aiello 2024 | Antonello2024 | Brosh-Nissimov 2024 | Gentile 2023 | Huang 2024 | Huygens 2023 | Longo 2023 | Marques 2024 | Meijer 2024 | Mikulska 2023 | Pasquini 2023 | Sanchez 2024 | Upasani 2023 |
| --- | --- | --- | --- | --- | --- | --- | --- | --- | --- | --- | --- | --- | --- |
| Representative study group (selection bias) | **High risk**  Only 3 cases with persistent COVID-19 | **High risk**  Only 9 cases with persistent COVID-19 | **High risk**  Only 14 cases with persistent COVID-19 | **High risk**  Only 4 cases with persistent COVID-19 | **High risk**  Only 6 cases with persistent COVID-19 | **High risk**  Only 6 cases with persistent COVID-19 | **High risk**  Only 15 cases with persistent COVID-19 | **High risk**  Only 5 cases with persistent COVID-19 | **High risk**  Only 15 cases with persistent COVID-19 | **High risk**  Only 22 cases with persistent COVID-19 | **High risk**  Only 14 cases with persistent COVID-19 | **High risk**  Only 10 cases with persistent COVID-19 | **High risk**  Only 9 cases with persistent COVID-19 |
| Outcome detectors blinded to intervention (detection bias): Objective outcomes (viral clearance, mortality) | **Low risk**  Not blinded, but unlikely to bias assessment of objective  outcomes | **Low risk**  Not blinded, but unlikely to bias assessment of objective  outcomes | **Low risk**  Not blinded, but unlikely to bias assessment of objective  outcomes | **Low risk**  Not blinded, but unlikely to bias assessment of objective  outcomes | **Low risk**  Not blinded, but unlikely to bias assessment of objective  outcomes | **Low risk**  Not blinded, but unlikely to bias assessment of objective  outcomes | **Low risk**  Not blinded, but unlikely to bias assessment of objective  outcomes | N/A | **Low risk**  Not blinded, but unlikely to bias assessment of objective  outcomes | **Low risk**  Not blinded, but unlikely to bias assessment of objective  outcomes | **Low risk**  Not blinded, but unlikely to bias assessment of objective  outcomes | **Low risk**  Not blinded, but unlikely to bias assessment of objective  outcomes | **Low risk**  Not blinded, but unlikely to bias assessment of objective  outcomes |
| Outcome detectors blinded to intervention (detection bias): Subjective outcomes (COVID-19 recurrence/relapse, adverse events, symptom resolution) | **High risk**  Not blinded and likely to bias assessment of subjective  outcomes | **High risk**  Not blinded and likely to bias assessment of subjective  outcomes | **High risk**  Not blinded and likely to bias assessment of subjective  outcomes | **High risk**  Not blinded and likely to bias assessment of subjective  outcomes | **High risk**  Not blinded and likely to bias assessment of subjective  outcomes | **High risk**  Not blinded and likely to bias assessment of subjective  outcomes | **High risk**  Not blinded and likely to bias assessment of subjective  outcomes | N/A | **High risk**  Not blinded and likely to bias assessment of subjective  outcomes | **High risk**  Not blinded and likely to bias assessment of subjective  outcomes | **High risk**  Not blinded and likely to bias assessment of subjective  outcomes | **High risk**  Not blinded and likely to bias assessment of subjective  outcomes | **High risk**  Not blinded and likely to bias assessment of subjective  outcomes |
| Complete outcome assessment/follow up (attrition bias): Viral clearance | **Low risk**  Assessed for all 3 cases | **Low risk**  Assessed for 8/9 cases. One case was not tested again, because infection was documented in BAL only. | **Low risk**  Assessed for all 14 cases | **Low risk**  Assessed for all 4 cases | **Low risk**  Assessed for all 6 cases | **Low risk**  Assessed for all 6 cases | **Low risk**  Assessed for all 15 cases | N/A | **Low risk**  Assessed for all 15 cases | **Low risk**  Assessed for all 22 cases | **Low risk**  Assessed for all 14 cases | **Low risk**  Assessed for all 10 cases | **Low risk**  Assessed for all 9 cases |
| Complete outcome assessment/follow up (attrition bias): COVID-19 recurrence/relapse | **Low risk**  Assessed for all 3 cases | N/A | **Low risk**  Assessed for all 14 cases | **Low risk**  Assessed for all 4 cases | **Low risk**  Assessed for all 6 cases | **Low risk**  Assessed for all 6 cases | **Low risk**  Assessed for all 15 cases | N/A | **Low risk**  Assessed for all 15 cases | **Low risk**  Assessed for all 22 cases | **Low risk**  Assessed for all 14 cases | **Low risk**  Assessed for all 10 cases | **Low risk**  Assessed for all 9 cases |
| Complete outcome assessment/follow up (attrition bias): All-cause mortality | N/A | **Low risk**  Assessed for all 9 cases | **Low risk**  Assessed for all 14 cases | **Low risk**  Assessed for all 4 cases | **Low risk**  Assessed for all 6 cases | **Low risk**  Assessed for all 6 cases | **Low risk**  Assessed for all 15 cases | N/A | **Low risk**  Assessed for all 15 cases | **Low risk**  Assessed for all 22 cases | **Low risk**  Assessed for all 14 cases | **Low risk**  Assessed for all 10 cases | **Low risk**  Assessed for all 9 cases |
| Complete outcome assessment/follow up (attrition bias): Mortality while SARS-CoV-2 positive | N/A | **Low risk**  Assessed for all 9 cases | **Low risk**  Assessed for all 14 cases | **Low risk**  Assessed for all 4 cases | **Low risk**  Assessed for all 6 cases | **Low risk**  Assessed for all 6 cases | **Low risk**  Assessed for all 15 cases | N/A | **Low risk**  Assessed for all 15 cases | **Low risk**  Assessed for all 22 cases | **Low risk**  Assessed for all 14 cases | **Low risk**  Assessed for all 10 cases | **Low risk**  Assessed for all 9 cases |
| Complete outcome assessment/follow up (attrition bias): Adverse events | **Low risk**  Assessed for all 3 cases | **Low risk**  Assessed for all 9 cases | N/A | N/A | N/A | N/A | **Low risk**  Assessed for all 15 cases | N/A | N/A | **Low risk**  Assessed for all 22 cases | **Low risk**  Assessed for all 14 cases | N/A | N/A |
| Complete outcome assessment/follow up (attrition bias): Symptom resolution | N/A | N/A | N/A | N/A | N/A | N/A | N/A | N/A | **Low risk**  Assessed for all 15 cases | N/A | **Low risk**  Assessed for all 14 cases | N/A | N/A |
| Well-defined study group (reporting bias) | **Unclear risk**  Characteristics of persistent cases not reported; interventions well described | **Low risk**  Cases and interventions well described | **Low risk**  Cases and interventions well described | **Unclear risk**  Characteristics of persistent cases not reported; interventions well described | **Low risk**  Cases and interventions well described | **Low risk**  Cases and interventions well described | **Low risk**  Cases and interventions well described | **Unclear risk**  No characteristics reported, interventions described | **Low risk**  Cases and interventions well described | **Low risk**  Cases and interventions well described | **Low risk**  Cases and interventions well described | **Low risk**  Cases and interventions well described | **Low risk**  Cases and interventions well described |
| Well-defined outcome (reporting bias): Viral clearance | **Low risk**  Persistent rRT-PCR with a Ct value < 26 at days 14, 20 and 33 | **Low risk**  Negative antigenic test or molecular test | **Low risk**  PCR test negative or Ct value > 30 at days 4-14 | **Low risk**  Cleared within 50 days | **Low risk**  Discharge with negative SARS-CoV-2 on pharyngeal swab | **Low risk**  Undetectable viral genome after 32 days | **Low risk**  Negativization within 16 days | N/A | **Low risk**  Full remission without any clinical or virological evidence of ongoing disease during last follow-up | **Low risk**  Alive and negative PCR at day 30 | **Low risk**  Viral clearance confirmed by PCR test | **Low risk**  Negative test at end of follow-up | **Low risk**  Reappearance of a positive test |
| Well-defined outcome (reporting bias): COVID-19 recurrence/relapse | N/A | N/A | **Low risk**  Clinical relapse with fever or dyspnea | **Low risk**  Persistence of infection | **Low risk**  Recurrence of fever | **Low risk**  Clearance after 257 days | **Low risk**  Relapse of viral infection | N/A | **Low risk**  Relapse/re-infection | **Low risk**  No response at day 30 | **Low risk**  No rebound (follow up median 26 days) | **Low risk**  Further complications | **Low risk**  Reappearance of a positive test |
| Well-defined outcome (reporting bias): All-cause mortality | N/A | **Low risk** | **Low risk** | **Low risk** | **Low risk** | **Low risk** | **Low risk** | N/A | **Low risk** | **Low risk** | **Low risk** | **Low risk** | **Low risk** |
| Well-defined outcome (reporting bias): Mortality while SARS-CoV-2 positive | N/A | **Low risk** | **Low risk**  One died due to clinical relapse | **Low risk**  Death with persistence of infection | **Low risk** | **Low risk** | **Low risk**  Death while SARS-CoV-2 positive | N/A | **Low risk** | **Low risk** Death while positive at day 62 | **Low risk** | **Low risk** | **Low risk** |
| Well-defined outcome (reporting bias): Adverse events | N/A | **Low risk** | N/A | N/A | N/A | N/A | **Low risk**  Severe adverse events | N/A | N/A | **Low risk**  Severe adverse events | **Low risk**  Major adverse events | N/A | N/A |
| Well-defined outcome (reporting bias): Symptom resolution | N/A | N/A | N/A | N/A | N/A | N/A | N/A | N/A | **Low risk**  Resolution of fever | N/A | **Low risk**  Resolution of COVID-19 | N/A | N/A |
| Well-defined risk estimates (analyses): Viral clearance | N/A | N/A | N/A | N/A | N/A | N/A | N/A | N/A | N/A | N/A | N/A | N/A | N/A |
| Well-defined risk estimates (analyses): COVID-19 recurrence/relapse | N/A | N/A | N/A | N/A | N/A | N/A | N/A | N/A | N/A | N/A | N/A | N/A | N/A |
| Well-defined risk estimates (analyses): All-cause mortality | N/A | N/A | N/A | N/A | N/A | N/A | N/A | N/A | N/A | N/A | N/A | N/A | N/A |
| Well-defined risk estimates (analyses): Mortality while SARS-CoV-2 positive | N/A | N/A | N/A | N/A | N/A | N/A | N/A | N/A | N/A | N/A | N/A | N/A | N/A |
| Well-defined risk estimates (analyses): Adverse events | N/A | N/A | N/A | N/A | N/A | N/A | N/A | N/A | N/A | N/A | N/A | N/A | N/A |
| Well-defined risk estimates (analyses): Symptom resolution | N/A | N/A | N/A | N/A | N/A | N/A | N/A | N/A | N/A | N/A | N/A | N/A | N/A |
| Important prognostic factors or follow-up taken adequately into account (confounding) | N/A | N/A | N/A | N/A | N/A | N/A | N/A | N/A | N/A | N/A | N/A | N/A | N/A |

Abbreviations: **N/A** not applicable

**Table S5: Search strategies nirmatrelvir/ritonavir**

| **Nirmatrelvir/ritonavir** |
| --- |
| **Database: Ovid MEDLINE(R) ALL 1946 to August 6, 2024**  # Searches  1 SARS-CoV-2/ or COVID-19/  2 ("2019 nCoV" or 2019nCoV or coronavir* or coronovir* or COVID or COVID19 or HCoV* or "nCov 2019" or "SARS CoV2" or "SARS CoV 2" or SARSCoV2 or "SARSCoV 2" or "severe acute respiratory syndrome coronavirus 2" or omicron* or omikron*).ti,ab.  3 or/1-2  4 ("PF‐07321332" or "PF 07321332" or "PF07321332" or paxlovid* or nirmatrelvir*).ti,ab,kf.  5 3 and 4  6 exp cohort studies/ or exp epidemiologic studies/ or exp clinical trial/ or exp evaluation studies as topic/ or exp statistics as topic/  7 ((control and study) or group* or (time and factors) or cohort or program or comparative stud* or evaluation studies or survey* or follow-up* or ci).mp.  8 or/6-7  9 (animals/ not humans/) or comment/ or editorial/ or exp review/ or meta analysis/ or consensus/ or exp guideline/  10 hi.fs. or case report.mp. or case report.pt.  11 or/9-10  12 8 not 11  13 5 and 12  14 limit 13 to yr="2022 -Current"  15 remove duplicates from 14 |
| **Database: Scopus (via Elsevier)**  TITLE-ABS ( "PF-07321332" OR "PF 07321332" OR "PF07321332" OR paxlovid* OR nirmatrelvir* ) AND TITLE-ABS ( ( ( control AND study ) OR group OR groups OR ( time AND factors ) OR program OR survey* OR cohort OR comparative AND stud* OR "evaluation studies" OR follow-up* ) ) AND PUBYEAR > 2021 AND PUBYEAR < 2025 AND NOT TITLE-ABS ( "case report" ) AND ( LIMIT-TO ( DOCTYPE , "ar" ) ) |

**Table S6: Search strategies remdesivir**

| **Remdesivir** |
| --- |
| **Database: Ovid MEDLINE(R) ALL 1946 to August 6, 2024**  # Searches  1 SARS-CoV-2/ or COVID-19/  2 ("2019 nCoV" or 2019nCoV or coronavir* or coronovir* or COVID or COVID19 or HCoV* or "nCov 2019" or "SARS CoV2" or "SARS CoV 2" or SARSCoV2 or "SARSCoV 2" or "severe acute respiratory syndrome coronavirus 2" or omicron* or omikron*).ti,ab.  3 or/1-2  4 (remdesivir* or GS5734 or "GS 5734" or "GS-5734" or veklury).ti,ab,kf.  5 3 and 4  6 exp cohort studies/ or exp epidemiologic studies/ or exp clinical trial/ or exp evaluation studies as topic/ or exp statistics as topic/  7 ((control and study) or group* or (time and factors) or cohort or program or comparative stud* or evaluation studies or survey* or follow-up* or ci).mp.  8 or/6-7  9 (animals/ not humans/) or comment/ or editorial/ or exp review/ or meta analysis/ or consensus/ or exp guideline/  10 hi.fs. or case report.mp. or case reports.pt.  11 or/9-10  12 8 not 11  13 5 and 12  14 limit 13 to yr="2022 -Current"  15 remove duplicates from 14 |
| **Database: Scopus (via Elsevier)**  Advanced search TITLE-ABS ( remdesivir* OR gs5734 OR "GS 5734" OR "GS-5734" OR veklury )  AND  TITLE-ABS ( covid OR covid19 OR "SARS-CoV-2" OR "SARS-CoV2" OR sarscov2 OR "SARSCoV-2" OR "SARS coronavirus 2" OR "2019 nCoV" OR "2019nCoV" OR "2019-novel CoV" OR "nCov 2019" OR "nCov 19" OR "severe acute respiratory syndrome coronavirus 2" OR "novel coronavirus disease" OR "novel corona virus disease" OR "novel coronavirus infection" OR "novel corona virus infection" OR "corona virus disease 2019" OR "coronavirus disease 2019" OR "novel coronavirus pneumonia" OR "novel corona virus pneumonia" OR "severe acute respiratory syndrome coronavirus 2" OR omicron* OR omikron* )  AND  TITLE-ABS ( ( ( control AND study ) OR group OR groups OR ( time AND factors ) OR program OR survey* OR cohort OR comparative AND stud* OR "evaluation studies" OR follow-up* ) )  AND PUBYEAR &GT; 2021 AND PUBYEAR &LT; 2025  AND NOT TITLE-ABS ( "case report" ) |

**Table S7: Search strategies ensitrelvir**

| **Ensitrelvir** |
| --- |
| **Database: Ovid MEDLINE(R) ALL 1946 to August 6, 2024**  # Searches  1 SARS-CoV-2/ or COVID-19/  2 ("2019 nCoV" or 2019nCoV or coronavir* or coronovir* or COVID or COVID19 or HCoV* or "nCov 2019" or "SARS CoV2" or "SARS CoV 2" or SARSCoV2 or "SARSCoV 2" or "severe acute respiratory syndrome coronavirus 2" or omicron* or omikron*).ti,ab.  3 or/1-2  4 (ensitrelvir* or xocova* or "S-217622" or "S217622" or "3bcl" or "3bclpro" or "3C like").ti,ab.  5 3 and 4  6 exp cohort studies/ or exp epidemiologic studies/ or exp clinical trial/ or exp evaluation studies as topic/ or exp statistics as topic/  7 ((control and study) or group* or (time and factors) or cohort or program or comparative stud* or evaluation studies or survey* or follow-up* or ci).mp.  8 or/6-7  9 (animals/ not humans/) or comment/ or editorial/ or exp review/ or meta analysis/ or consensus/ or exp guideline/  10 hi.fs. or case report.mp. or case reports.pt.  11 or/9-10  12 8 not 11  13 5 and 12  14 limit 13 to yr="2022 -Current"  15 remove duplicates from 14 |
| **Database: Scopus (via Elsevier)**  Advanced search TITLE-ABS ( ensitrelvir* OR xocova* OR "S-217622" OR "S217622" OR "3CL protease" OR "3CL inhibitor" OR "3CLpro protease" OR "3CLpro inhibitor" OR "3C like protease" OR "3C like inhibitor" ) AND TITLE-ABS ( ( control AND study ) OR group OR groups OR ( time AND factors ) OR program OR survey* OR cohort OR comparative AND stud* OR "evaluation studies" OR follow-up* ) AND PUBYEAR > 2021 AND PUBYEAR < 2025 AND NOT TITLE-ABS ( "case report" ) AND ( LIMIT-TO ( DOCTYPE , "ar" ) ) |

**Table S8: Search strategies molnupiravir**

| **Molnupiravir** |
| --- |
| **Database: Ovid MEDLINE(R) ALL 1946 to August 6, 2024**  # Searches  1 SARS-CoV-2/ or COVID-19/  2 ("2019 nCoV" or 2019nCoV or coronavir* or coronovir* or COVID or COVID19 or HCoV* or "nCov 2019" or "SARS CoV2" or "SARS CoV 2" or SARSCoV2 or "SARSCoV 2" or "severe acute respiratory syndrome coronavirus 2" or omicron* or omikron*).ti,ab.  3 or/1-2  4 (molnupiravir* or "MK-4482" or "MK 4482" or MK4482* or "EIDD-2801" or EIDD2801* or "EIDD-1931" or EIDD1931* or lagevrio*).ti,ab,kf,nm.  5 3 and 4  6 Virus Shedding/  7 Viral Load/  8 ((viral* adj2 (burden* or load*)) or (virus adj2 titer*)).ti,ab,kf.  9 (Viral shedding or shedding median or IQR).ti,ab,kf.  10 (virologic* adj2 clear*).ti,ab,kf.  11 (virologic* adj4 response).ti,ab.  12 (persisten* or prolong* or pro-long*).ti,ab.  13 or/6-12  14 3 and 4 and 13  15 exp cohort studies/ or exp epidemiologic studies/ or exp clinical trial/ or exp evaluation studies as topic/ or exp statistics as topic/  16 ((control and study) or group* or (time and factors) or cohort or program or comparative stud* or evaluation studies or survey* or follow-up* or ci).mp.  17 or/15-16  18 (animals/ not humans/) or comment/ or editorial/ or exp review/ or meta analysis/ or consensus/ or exp guideline/  19 hi.fs. or case report.mp.  20 or/18-19  21 17 not 20  22 3 and 4 and 13 and 21  #15 - #21: controlled NRS filter – high sensitivity, 92.17 sens Waffenschmidt, Siw et al. “Development and validation of study filters for identifying controlled non-randomized studies in PubMed and Ovid MEDLINE.” Research synthesis methods vol. 11,5 (2020): 617-626. doi:10.1002/jrsm.1425 |
| **Database: Scopus (via Elsevier)**  Advanced search TITLE-ABS ( covid OR covid19 OR "SARS-CoV-2" OR "SARS-CoV2" OR sarscov2 OR "SARSCoV-2" OR "SARS coronavirus 2" OR "2019 nCoV" OR "2019nCoV" OR "2019-novel CoV" OR "nCov 2019" OR "nCov 19" OR "severe acute respiratory syndrome coronavirus 2" OR "novel coronavirus disease" OR "novel corona virus disease" OR "novel coronavirus infection" OR "novel corona virus infection" OR "corona virus disease 2019" OR "coronavirus disease 2019" OR "novel coronavirus pneumonia" OR "novel corona virus pneumonia" OR "severe acute respiratory syndrome coronavirus 2" )  AND TITLE-ABS ( molnupiravir* OR "MK-4482" OR "MK 4482" OR mk4482* OR "EIDD-2801" OR eidd2801* OR "EIDD-1931" OR eidd1931* OR lagevrio* )  AND TITLE-ABS ( ( virologic* W/2 clear* ) OR ( virologic* W/4 response ) OR ( viral* W/2 ( burden* OR load* ) ) OR ( virus W/2 titer* ) OR "viral shedding" OR "shedding median" OR iqr OR persisten* OR prolong* OR pro-long* )  AND TITLE-ABS ( ( control AND study ) OR group* OR ( time AND factors ) OR cohort OR program OR comparative AND stud* OR evaluation AND studies OR survey* OR follow-up* OR ci )  AND PUBYEAR > 2020 AND PUBYEAR < 2025  AND ( LIMIT-TO ( DOCTYPE , "ar" ) ) |

**Table S9: Study characteristics**

| **Study** | **Study design** | **Number of participants (total)** | **Number of persistent cases** | **Follow-up** | **Recruitment period** | **Country** | **Funding** | **Conflicts of interest** |
| --- | --- | --- | --- | --- | --- | --- | --- | --- |
| Aiello 2024 | prospective cohort study | 83 | 3 | after 5 days of antiviral treatment | remdesivir: December 2021 to April 2022 nirmatrelvir/ritonavir: May 2022 to August 2022 | Spain | NR | CG-V, AS, JM, OP have received honoraria for talks or grant support from pharmaceutical companies. |
| Antonello 2024 | retrospective study | 9 | 9 | the next day after the end of the combination treatment, and after the radiological control (day 5 to day 105) | December 2022 to April 2023 | Italy | NR | No competing interests |
| Brosh-Nissimov 2024 | retrospective study | 14 | 14 | median follow-up 45 days (IQR 12-89) | March 2022 to not reported | Israel | "This research did not receive any specific grant from funding agencies in the public, commercial, or not-for-profit sectors." | TBN received honoraria and consultation fees from pharmaceutical companies. |
| Huang 2024 | case series | 6 | 6 | individual case descriptions: until fever diminished, up to several months | December 2022 to July 2023 | China | "This work was supported by the National High Level Hospital Clinical Research Funding (BJ-2023-159 and BJ-2021-189)." | No competing interests |
| Huygens 2023 | case series / retrospective cohort | 6 | 6 | up to negative tests |  | Netherlands | "This study has been partly funded by EU Horizon 2020 projects RECoVer (grant number: 101003589) and VEO (grant number: 874735)." | BOM has received public funding. All other authors declare to have no competing interests |
| Longo 2023 | case series | 15 | 15 | up to 90 days | March 2022 to February 2023 | Italy | "This research received no external funding." | No competing interests |
| Meijer 2024 | case series / retrospective study | 15 | 15 | at least three months | March 2022 to May 2022 | Israel | "No funding was received for this study" | No competing interests |
| Mikulska 2023 | retrospective study | 22 | 22 | 63 days (IQR 47 to 104) after the first combination treatment and 21 days (IQR 6 to 58) after the second treatment in 4 cases | 21 February 2020 to 7 October 2022 | Italy | NR | EA , MB, DRG report research grants and/or personal fees from pharmaceutical companies. All other authors declare to have no competing interests |
| Pasquini 2023 | case series | 14 | 14 | up to 30 days after the end of treatment | November 2022 to February 2023 | Italy | "no external funding was received for this study" | No competing interests |
| Gentile 2023 | restrospective study | 11 | 4 | median of 44 (IQR 10 to 92) days after the administration of combination therapy | April 2023 to June 2023 | Italy | "This research was supported by EU funding within the NextGenerationEUMUR PNRR Extended Partnership initiative on Emerging Infectious Diseases (project no. PE00000007, INF-ACT) and POR Campania FESR 2014–2020—Asse 1—Obiettivo Specifico 1.3.—Azione 1.3.1." | No competing interests |
| Sanchez 2024 | cases from retrospective cohort | 93 | 5 | as long as positive | March 2022 to November 2022 | USA | " National Institute of Allergy and Infectious Diseases of the National Institutes of Health under award number T32AI118690 and National Institutes of Health/ National Cancer Institute (NIH/ NCI) Cancer Center support grants P30 CA15704" | SAP received research support from a pharmaceutical company and participated in clinical trials. CL was site investigator for a pharmaceutical company. All other authors declare to have no competing interests |
| Marques 2024 | retrospective study | 5 | 5 | as long as necessary | May 2020 to January 2023 | USA | "This work was supported in part by the Penn University Research Foundation. Funding was provided by a contract award from the Centers for Disease Control and Prevention (CDC BAA 200–2021-10986 and 75D30121C11102/000HCVL1-2021-55232), the Penn Center for Global Genomics and Health Equity Keystone Pilot Grant (GGHE-KP-2021–001), philanthropic donations to the Penn Center for Research on Coronaviruses and Other Emerging Pathogens, and in part by NIH grant R61/33-HL137063 and AI140442-supplement for SARS-CoV-2. B.J.K. was supported by NIH K23 AI 121485. Additional assistance was provided by the Penn Center for AIDS Research (P30-AI045008)" | No competing interests |
| Upasani 2023 | Case series for the persistent cases | 9 | 9 | up to consistent negative testing, between 63 and 982 days post-infection | July and August 2022 | UK | "Financial support. R. C. L. B. reports intramural funding from the Francis Crick Institute." | AT, SW, AS, SW, DML, RCLB, ST received personal fees or payment for advisory board attendance or grant support or travel support from pharmaceutical companies. All other authors declare to have no competing interests |

**Table S10: Participant characteristics**

| **Study** | **Population** | **Previous COVID-19 treatment** | **age (median, in years (IQR))** | **sex (% female)** | **Vaccination status** | **COVID-19 prophylaxis** | **Obesity (%)** | **Diabetes (%)** | **Respiratory disease (%)** | **Hypertension (%)** | **Kidney disease** |
| --- | --- | --- | --- | --- | --- | --- | --- | --- | --- | --- | --- |
| Aiello 2024 | Those with persistence AND hospitalised from immunocompromised patients from a consecutive cohort | 3/3 N/R | RMV: 58 (46 to 65) N/R: 67 (IQR 59 to 74) | Remdesivir: 35.7% Nirmatrelvir/ritonavir: 36.6% | prior COVID-19 vaccination Remdesivir: 39/42 Nirmatrelvir/ritonavir: 39/41  minimum of 3 doses: Remdesivir: 45.2% Nirmatrelvir/ritonavir: 85.4% | NR | NR | Remdesivir: 9.5% Nirmatrelvir/ritonavir: 14.6% | Remdesivir: 9.5% Nirmatrelvir/ritonavir: 7.3% | Remdesivir: 31% Nirmatrelvir/ritonavir: 36.6% | chronic renal failure Remdesivir: 11.9% Nirmatrelvir/ritonavir: 12.2% |
| Antonello 2024 | Patients with haematological malignancies:  - 3 FL  - 2 NHL  - 1 CLL  - 1 AML  - 1 CLL, FL | 1/9 N/R + tixagevimab/cilgavimab 1/9 N/R + MOL 2/9 MOL + sotrovimab 1/9 tixagevimab/cilgavimab 2/9 RDV 2/9 N/R | median 68 (IQR 63.5 to 74.5) years | 33% | 2/9 received 5 doses 3/9 received 4 doses 4/9 received 3 doses | NR | NR | NR | NR | NR | NR |
| Brosh-Nissimov 2024 | Severely immunocompromised patients with pCOVID-19 - 2 kidney transplant - 11 B-cell lymphoproliferative disease (7 NHL, 4 CLL) receiving BCDT - 1 rheumatoid arthritis treated with BCDT | not reported | 74 years (IQR 69 to 79) | 36% | 9/14 received 4 doses at a median of 146 (IQR 119,244) days before disease onset 1/14 received 3 doses 262 days before disease onset 2 received 2 doses >1 year before 2 unvaccinated | 3/14 received tixegavimab/cilgavimab 86, 104 and 114 days before DO (150/150 mg) | 14% | 43% | 21% | 57% | chronic renal failure:  4/14 (28.5%) |
| Huang 2024 | Immunocompromised individuals with recurrent fever caused by SARS-CoV-2  persistent negative upper respiratory tract test, positive lower respiratory tract test  - 6 FL | not reported |  | 33,33% | 6 had at least one history of SARS-CoV-2 infection 1 vaccinated | NR, probably none | NR | 50% | NR | NR | 1 /6 CKD (17%) 2 HepB |
| Huygens 2023 | Immunocompromised individuals with a prolonged SARS-CoV-2 infection (positive PCR with Ct values <30 for ≥20 days)  - 4 B-cell malignancy  - 1 T-cell malignancy  - 1 common variable immune deficiency | 1/6 tixagevimab/cilgavimab 2/6 CP 2/6 tixagevimab/cilgavimab + CP 1/6 none | median age 58 years (range 44–70 years) | 66,67% | 6/6 at least 2 vaccination doses | NR, probably none | NR | NR | NR | NR | NR |
| Longo 2023 | Immunocompromised patients with persistent SARS-CoV-2  - 11 onco-haematological disease (8 NHL, 1 HL, 1 CLL, 1 hypogammaglobulinemia)  - 4 HIV/AIDS | 6/15 N/R 4/15 RMV 1/15 sotrovimab 4/15 none | median age 65 (IQR 58.3–73) | 40% | median number of vaccine doses: 3 (IQR 1-5) | prophylactic mAbs: 27% | 13% | 27% | 13% |  | NR |
| Meijer 2024 | Immunocompromised patients with persistent SARS-CoV-2 - 11 onco-haematological disease (8 NHL, 1 HL, 1 CLL, 1 hypogammaglobulinemia) - 4 HIV/AIDS | 7/15 N/R 2/15 RMV 1/15 MOL  1/15 N/R then RMV 4/15 none | 74 (67 to 78) | 47% | 14/15 fully vaccinated (3 or more doses of Pfizer BioNTech) | NR | NR | NR | NR | NR | NR |
| Mikulska 2023 | Immunocompromised patients with prolonged/relapsed COVID-19  - 15 NHL, 3 AML, 1 CLL  - 2 renal transplant  - 1 severe hypogammaglobulinemia | 5/22 MOL 3/22 N/R 1/22 RMV 3/22 sotrovimab 1/22 bamlanivimab/casirivimab 9/22 none | 70 (55 to 75) | 41% | median number of doses 3 (min 1; max 4) | 1/22 tixagevimab/cilgavimab | 4.50% | 13.60% | NR | NR | chronic renal failure: 9% |
| Pasquini 2023 | Patients with impaired adaptive humoral immunity and SARS-CoV-2 persistent infection  - 12 B-cell lymphoma, 1 CLL  - 1 MS treated with ocrelizumab | 6/14 N/R 1/14 MOL 1/14 sotrovimab 6/14 none | 60.0 (56.25 to 68.5) | 21,40% | 3 doses: 7/14 4 doses: 6/14 5 doses: 1/14 | 2/14 received tixagevimab/cilgavimab as PreP and 2/14 after acute infection | NR | NR | NR | NR | NR |
| Gentile 2023 | Immunocompromised patients with prolonged/relapsed COVID-19 - 15 NHL, 3 AML, 1 CLL - 2 renal transplant  - 1 severe hypogammaglobulinemia | 1/4 RMV 3/4 RMV + dexamethasone | early treatment: 52 (35 to 56) late treatment: 63 (42 to 77) | early treatment: 43% late treatment: 50% | - Vaccinated early treatment: 71% late treatment: 75% - Vaccine doses early treatment: median 3 (IQR 3) late treatment: median 3 (IQR 3) | tixagevimab-cilgavimab: early treatment: 1/7 late treatment: 0/4 | NR | NR | NR | NR | NR |
| Sanchez 2024 | 10 patients with pCOVID-19  - 3 B-ALL, 1 myeloid sarcoma, 1 lymphoma, 3 AML, 1 CLL, 1 MM | 5/5 bebtelovimab | range: 38 to 79 | 40% | 0 doses: 1 2 doses: 3 3 doses: 4 4 doses: 2 | NR | NR | NR | NR | NR | NR |
| Marques 2024 | Immunocompromised patients with pCOVID-19 (>21 days viral shedding)  - 4 lymphoma  - 1 transplant | NR | NR | NR | NR | NR | NR | NR | NR | NR | NR |
| Upasani 2023 | 9 immunosuppressed patients with chronic/PCR–positive SARS-CoV-2 infection who received immunoglobulin therapy during their treatment for COVID-19 | not reported | range: 50-84 | 67% | all received at least 2 doses | NR | NR | NR | NR | NR | NR |

**Table S11: Characteristics of viral persistence**

| **Study** | **Description of viral persistence** | **Symptoms** | **Time to presentation of**  **pCOVID-19** | **Treatment to treat pCOVID-19** |
| --- | --- | --- | --- | --- |
| Aiello 2024 | persistent Ct < 26 or positive sgRNA at the end of the treatment regimen | NR | Day 14, day 20 and day 33 after infection | 2/3 remdesivir (5 days) + hyperimmune plasma 1/3 remdesivir (5 days), hyperimmune plasma + sotrovimab |
| Antonello 2024 | history of clinical and/or virological failure with antiviral monotherapy against SARS-CoV-2 | 5/9 fever, fatigue  3/9 cough  2/9 dyspnea  1/9 sore throat  1/9 asymptomatic | Median time between initial diagnosis and combination treatment 69 days (IQR 32–  193.5 days, range 20–272 days) | 4/9 N/R (150-300/100 mg; 10 days) + molnupiravir (800 mg; 10 days)  1/9 N/R (150/100 mg; 10 days) + remdesivir (200 mg LD, 100 mg q24h; 10 days)  1/9 remdesivir (200 mg LD,100mg q24; 10 days) + molnupiravir (800 mg; 10 days) + sotrovimab (500mg)  2/9 N/R (150-300/100 mg; 10 days) + remdesivir (200 mg LD, 100 mg q24h; 10 days) + sotrovimab (500mg)  1/9 N/R (150-300/100 mg; 10 days) + remdesivir (200 mg LD, 100 mg q24h; 10 days) + molnupiravir (800 mg q12h; 10 days at days 11-21) |
| Brosh-Nissimov 2024 | prolonged symptomatic infection and lack of serologic responses | significant symptoms:  cough: 11  fatigue: 11  fever: 10  dyspnea: 5  weight loss: 4  diarrhea: 4  hypoxemia: 9 | Median time from disease onset to presentation: 20 days (IQR 15,39) | 14/14 remdesivir (5-day course) 11/14 N/R (5-day course) 1/14 molnupiravir (duration not reported) Intramuscular tixagavimab/cilgavimab: 100% dexamethasone 6mg for 5-10 days: 12 |
| Gentile 2023 | prolonged or relapsing symptoms and persistent SARS-CoV-2 RTPCR on respiratory samples in the absence of serological response to the infection after 14 days | NR | Median of 79 (IQR 48–112) days | 4/4 combination antiviral treatment with 10 days remdesivir + 5 days N/R |
| Huang 2024 | persistent negative testingbefore hospitalisation (reception of antibacterial and antifungals)  Positive on BALF mNGS test, SARS-CoV-2 identified | persistent fever  shortness of breath  cough  dyspnea | Time between positive test and presentation with recurrent fever but negative test at this hospital between 1 and 6 months | 6/6 N/R (immediately) p1: 5 days N/R, again N/R 1day 2x (2 months after) p2: 5 days N/R; again 5 days N/R, methylprednisolone p3: 5 days N/R, 5 days molnupiravir, methylprednisolone, prednisolone p4: 5 days N/R, 11 days methylprednisolone; again N/R p5: 5 days N/R, 5 days methylprednisolone p6: 5 days N/R, 3 days prednisone |
| Huygens 2023 | positive PCR with Ct values <30 for ≥20 days | NR | Median time between first positive SARS-CoV-2 test and start of nirmatrelvir/ritonavir was 70 days (range 20–231 days) | 6/6 N/R (5-day course)  + 2 units of 300 mL of high-titer CP with a BA.5 neutralizing titer of 1/640 |
| Longo 2023 | No negativisation after early therapy administration | NR | Median time from the first positive swab to the rescue therapy for persistent SARS-CoV-2 infection was 28 days (IQR 24.5–44.5) | 6/15 one antiviral: remdesivir (3-5 days), N/R (5-10 days), or molnupiravir  5/15 sequential treatment 2/15 one antiviral + one mAb, depending on course  2/15 combination N/R (10 or 20 days) + remdesivir (10 days) |
| Marques 2024 | >21 days positive by the nucleic acid amplification test | NR | > 21 days | 4/5 remdesivir; one received 4 courses (earlier variant), and three only 1 course (no duration) + 1 bebtelovimab + 1 sotrovimab + 3 received dexamethasone |
| Meijer 2024 | Signs and symptoms of COVID-19 (fever ≥38.0 ◦C or new respiratory symptoms), for at least 14 days after the initial diagnosis; Evidence suggesting high viral load: a positive RT-PCR test for SARSCoV-2 on nasopharyngeal swab or broncho-alveolar lavage fluid with a cycle threshold (CT) value < 30, or a positive SARS-CoV-2 antigen test | 5/15 fever  4/15 respiratory symptoms  6/15 fever + respiratory symptoms | Median symptom duration at initiation of therapy: 32 (IQR 29 to 47) | 15/15 combination of N/R 300/100 mg PO and remdesivir 200 mg IV on day 1, followed by 100 mg/day for a total of five days + 8/15 corticosteroids + 3/15 tocilizumab + 6/15 tixagevimab/cilgavimab |
| Mikulska 2023 | no negative swab; and negative nasopharyngeal swab but BALF positive for SARS-CoV-2 and no other pathogen? | NR | Median time from SARS-CoV-2 diagnosis to the first combination therapy was 42 days (IQR 29 to 100) | 18/22 triple combination therapy: 2 antivirals + mAbs - 3 sotrovimab - 15 tixagevimab/cilgavimab  4/22 combination therapy with 2 antivirals only - 20/22 remdesivir (10-days) and N/R (5 days) - 2/22 remdesivir (10 days) and molnupiravir (5 days) |
| Pasquini 2023 | defined by a positive RT-PCR, including nasopharyngeal swab (NPS) or lower respiratory tract sample, with  radiological and/or clinical signs of infection after at least 21 days from the first Sars-CoV-2 positive test | 2 persisting cough  9 signs of interstitial pneumonitis | Median time between diagnosis of SARS‐CoV‐2 infection and initiation of combination therapy: 42.0 days (IQR 35.0–45.7) | 14/14 combination antiviral therapy with remdesivir (200 mg first day, then 100 mg daily) and N/R (300/100)  - 10/14 patients received a 10-day treatment cycle - 3/14 patients had longer courses of 13, 22, and 12 days (due to persistence of PCR test positivity - 1/14 discontinued treatment after 5 days because viral clearance was confirmed after 3 days of treatment |
| Sanchez 2024 | positive PCR test beyond day 30 | NR | > 30 days | Those positive after day 60 received treatment: 1/5 remdesivir 2/5 N/R 2/5 no further treatment |
| Upasani 2023 | chronic/persistent polymerase chain reaction (PCR)–positive SARS-CoV-2 infection | NR | Between 23 and 901 days post-infection onset | All participants received IVIG as experimental strategy. Previous number of courses of Remdesivir (duration of a course not reported): 1 course: 6 2 courses: 2 3 courses: 1 |

**Table S12: Outcomes - combination therapy with at least two direct antiviral agents**

| **Study ID** | **Population** | **N combination of at least two direct antiviral agents** | **Treatment to treat viral persistence** | **Cleared at first attempt** | **recurrence/relapse** | **death** | **death of SARS-CoV-2 / while positive** | **Adverse events** | **Symptom resolution** |
| --- | --- | --- | --- | --- | --- | --- | --- | --- | --- |
| Antonello 2023 | Patients with haematologic malignancies 3/9 FL 2/9 NHL 1/9 CLL 1/9 AML 1/9 CLL, FL | 9 | 5/9 two antivirals 4/9 nirmatrelvir/ritonavir (150-300/100 mg; 10 days) + molnupiravir (800 mg; 10 days) 1/9 nirmatrelvir/ritonavir (150/100 mg; 10 days) + remdesivir (200 mg LD, 100 mg q24h; 10 days)  4/9 three antivirals 1/9 remdesivir (200 mg LD,100mg q24; 10 days) + molnupiravir (800 mg; 10 days) + sotrovimab (500mg) 2/9 nirmatrelvir/ritonavir (150-300/100 mg; 10 days) + remdesivir (200 mg LD, 100 mg q24h; 10 days) + sotrovimab (500mg) 1/9 nirmatrelvir/ritonavir (150-300/100 mg; 10 days) + remdesivir (200 mg LD, 100 mg q24h; 10 days) + molnupiravir (800 mg q12h; 10 days at days 11-21) | 8 | 0 | 1 | 0 | 2 | NR |
| Huang 2024 | immunocompromised individuals with recurrent fever caused by SARS-CoV-2 persistent negative upper respiratory tract test, positive lower respiratory tract test - 6 follicular lymphpma | 1 | 6/6 Nirmatrelvir/ritonavir (immediately) p1: 5 day nirmatrelvir/ritonavir, again nirmatrelvir/ritonavir 1day 2x (2 months after) p2: 5 day nirmatrel/ritonavir, moxifloxacin; again 5 day nirmatrelvir/ritonavir, methylprednisolone p3: 5 day nirmatrelvir/ritonavir, 5 day molnupiravir, methylprednisolone, prednisolone p4: 5 day nirmatrelvir/ritonavir, 11 day azvudine, methylprednisolone; again nirmatrelvir/ritonavir p5: 5 day nirmatrelvir/ritonavir, 5 day methylprednisolone p6: 5 day nirmatrelvir/ritonavir, ceftriaxone 3day, prednisone | 1 | 0 | 0 | 0 | NR | NR |
| Longo 2023 | immunocompromised patients with persistent SARS-CoV-2 - 11 onco-hematological disease (8 NHL, 1 HL, 1 CLL, 1 Hypogammaglobulinemia) - 4 HIV/Aids | 2 | 12/15 one antiviral: remdesivir (9p) (3-5 days), nirmatrelvir/ritonavir (2 p) (5-10 days), or molnupiravir (1 p)  1/15 Combi DAA + mAb  2/15 Combi 2 DAA | 2 | 0 | 1 | 0 | 1 | NR |
| Meijer 2024 | immunocompromised haemato-oncological patients  - 4/15 CLL - 4/15 MM - 7/15 Malignant lymphoma - 12/15 Hypogammaglobulinemia | 15 | 15/15 combination of nirmatrelvir/ritonavir 300/100 mg PO and remdesivir 200 mg IV on day 1, followed by 100 mg/day for a total of five days + 8/15 corticosteroids + 3/15 tocilizumab + 6/15 tixagevimab/cilgavimab | 9 | 4 | 0 | 0 | NR | median 3 (IQR 1-3) days |
| Mikulska 2023 | immunocompromised patients with prolonged/relapsed COVID-19 - 15 NHL - 3 AML - 1 CLL - 2 renal transplant  - 1 severe hypogammaglobulinemia | 22 | 18/22 triple combination therapy: 2 antivirals + mAbs - 3 sotrovimab - 15 tixagevimab/cilgavimab  4/22 combination therapy with 2 antivirals only - 20/22 remdesivir (10-days) and nirmatrelvir/ritonavir (5 days) - 2/22 remdesivir (10 days) and molnupiravir (5 days) | 16 | 6 | 3 | 3 | 2 | NR |
| Pasquini 2023 | Patients with impaired adaptive humoral immunity and SARS-CoV-2 persistent infection - 12 B-cell lymphoma - 1 CLL - 1 MS treated with ocrelizumab | 14 | 14/14 combination antiviral therapy with remdesivir (200 mg first day, then 100 mg daily) and nirmatrelvir/ritonavir (300/100 mg bid, or 150/100 mg in case of mild kidney impairment with estimated glomerular filtration rate (eGFR) <60 mL/min) - 10/14 patients received a 10 day treatment cycle - 3/14 patients had longer courses of 13, 22, and 12 days (due to persistence of PCR test positivity - 1/14 discontinued treatment after 5 days because viral clearance was confirmed after 3 days of treatment | 14 | 0 | 0 | 0 | 0 | median 6 days (IQR 4.2 to 10.7) |
| Gentile 2023 | 4 immunocompromised patients - 2 NHL - 1 MM - 1 other immunodeficiency | 4 | 4/4 combination antiviral treatment with 10 days remdesivir + 5 days N/R | 3 | 1 | 1 | 1 | NR | NR |

**Table S13: Outcomes - combination therapy with one direct antiviral agent and passive immunisation**

| **Study ID** | **Population** | **N combination with one direct antiviral agent and passive immunisation** | **Treatment to treat viral persistence** | **Cleared at first attempt** | **recurrence/relapse** | **death** | **death of SARS-CoV-2 / while positive** | **Adverse events** | **Symptom resolution** |
| --- | --- | --- | --- | --- | --- | --- | --- | --- | --- |
| Aiello 2023 | those with persistence AND hospitalised from immunocompromised patients from a cohort of consecutive patients | 3 | 2/3 remdesivir (5 days) + hyperimmune plasma 1/3 remdesivir (5 days), hyperimmune plasma + sotrovimab | 3 | 0 | 0 | 0 | 0 | NR |
| Huygens 2023 | immunocompromised individuals with a prolonged SARS-CoV-2 infection (positive PCR with Ct values <30 for ≥20 days) - 4 B-cell malignancy - 1 T-cell malignancy - 1 common varialbe immune deficiency | 6 | 6/6 nirmatrevir/ritonavir (5 day course)  + 2 units of 300 mL of high-titer CP with a BA.5 neutralizing titer of 1/640 | 5 | 1 | 0 | 0 | NR | NR |
| Longo 2023 | immunocompromised patients with persistent SARS-CoV-2 - 11 onco-hematological disease (8 NHL, 1 HL, 1 CLL, 1 Hypogammaglobulinemia) - 4 HIV/Aids | 1 | 12/15 one antiviral: remdesivir (9p) (3-5 days), nirmatrelvir/ritonavir (2 p) (5-10 days), or molnupiravir (1 p)  1/15 Combi DAA + mAb  2/15 Combi 2 DAA | 1 | 0 | 0 | 0 | 0 | NR |
| Upasani 2023 | 9 immunosuppressed patients with chronic/persistent polymerase chain reaction (PCR)–positive SARS-CoV-2 infection who received immunoglobulin therapy during their treatment for COVID-19 - 5 FL - 1 CLL - 2 rheumatoid arthritis - 1 MALT lymphoma | 9 | All participants received IVIG as experimental strategy. Previous number of courses of Remdesivir (duration of a course not reported): 1 course: 6 2 courses: 2 3 courses: 1 | 8 | 1 | 0 | 0 | NR | NR |

**Table S14: Outcomes - monotherapy including a single direct antiviral agent**

| **Study ID** | **Population** | **N monotherapy** | **Treatment to treat viral persistence** | **Cleared at first attempt** | **recurrence/relapse** | **death** | **death of SARS-CoV-2 / while positive** | **Adverse events** | **Symptom resolution** |
| --- | --- | --- | --- | --- | --- | --- | --- | --- | --- |
| Huang 2024 | immunocompromised individuals with recurrent fever caused by SARS-CoV-2 persistent negative upper respiratory tract test, positive lower respiratory tract test - 6 follicular lymphpma | 5 | 6/6 Nirmatrelvir/ritonavir (immediately) p1: 5 day nirmatrelvir/ritonavir, again nirmatrelvir/ritonavir 1day 2x (2 months after) p2: 5 day nirmatrel/ritonavir, moxifloxacin; again 5 day nirmatrelvir/ritonavir, methylprednisolone p3: 5 day nirmatrelvir/ritonavir, 5 day molnupiravir, methylprednisolone, prednisolone p4: 5 day nirmatrelvir/ritonavir, 11 day azvudine, methylprednisolone; again nirmatrelvir/ritonavir p5: 5 day nirmatrelvir/ritonavir, 5 day methylprednisolone p6: 5 day nirmatrelvir/ritonavir, ceftriaxone 3day, prednisone | 5 | 3 | 0 | 0 | NR | NR |
| Longo 2023 | immunocompromised patients with persistent SARS-CoV-2 - 11 onco-hematological disease (8 NHL, 1 HL, 1 CLL, 1 Hypogammaglobulinemia) - 4 HIV/Aids | 12 | 12/15 one antiviral: remdesivir (9p) (3-5 days), nirmatrelvir/ritonavir (2 p) (5-10 days), or molnupiravir (1 p)  1/15 Combi DAA + mAb  2/15 Combi 2 DAA | 12 | 0 | 1 | 1 | 0 | NR |
| Sanchez 2024 | 5 patients with persistent COVID-19 - 3 B-ALL - 1 Lymphoma - 1 AML | 3 | Those positive after day 60 received treatment: 1/5 remdesivir 2/5 nirmatrelvir/ritonavir 2/5 no further treatment | 3 | 0 | 0 | 0 | NR | NR |

**Table S15: Sensitivity analysis: combination therapy with one direct antiviral agent and polyclonal antibodies versus combination therapy with one direct antiviral agent and monoclonal antibodies**

| **Outcome** | **Study** | **n** | **N** |
| --- | --- | --- | --- |
| **Viral Clearance** | **Treatment with polyclonal antibodies** | | |
|  | Upasani 2023 | 8 | 9 |
|  | Huygens 2023 | 5 | 6 |
|  | Aiello 2024 | 2 | 2 |
|  |  | 15 | 17 |
|  | **Treatment with monoclonal antibodies** | | |
|  | Longo 2023 | 1 | 1 |
|  | Aiello 2024 | 1 | 1 |
|  |  | 2 | 2 |
| **Recurrence/relapse** | **Treatment with monoclonal antibodies** | | |
|  | Upasani 2023 | 1 | 9 |
|  | Huygens 2023 | 1 | 6 |
|  | Aiello 2024 | 0 | 2 |
|  |  | 2 | 17 |
|  | **Treatment with mAbs** | | |
|  | Longo 2023 | 0 | 1 |
|  | Aiello 2024 | 0 | 1 |
|  |  | 0 | 2 |
| **Mortality** | **Treatment with polyclonal antibodies** | | |
|  | Upasani 2023 | 0 | 9 |
|  | Huygens 2023 | 0 | 6 |
|  | Aiello 2024 | 0 | 2 |
|  |  | 0 | 17 |
|  | **Treatment with monoclonal antibodies** | | |
|  | Longo 2023 | 0 | 1 |
|  | Aiello 2024 | 0 | 1 |
|  |  | 0 | 2 |
